# Causal relationships between irritable bowel syndrome and brain functional networks: A Mendelian randomization study

**DOI:** 10.1515/jtim-2026-0047

**Published:** 2026-06-13

**Authors:** Ruqiao Duan, Tao Zhang, Siqi Lu, Lingling Zhu, Qi Zhang, Yongmei Bai, Kun Wang, Liping Duan

**Affiliations:** Department of Gastroenterology, Peking University Third Hospital, Beijing, China; Beijing Key Laboratory for Helicobacter pylori Infection and Upper Gastrointestinal Diseases, Beijing, China; Department of Gastroenterology, Beijing Tsinghua Changgung Hospital, Beijing, China

**Keywords:** irritable bowel syndrome, brain resting-state functional magnetic resonance imaging, causal relationships, Mendelian randomization

## Abstract

**Background and Objective:**

The association between irritable bowel syndrome (IBS) and brain function remains unclear. We conducted Mendelian randomization (MR) analysis to investigate the possible causal relationships between them.

**Methods:**

We performed bidirectional MR analysis using genome-wide association study data from IBS and 191 brain resting-state functional magnetic resonance imaging (rsfMRI) traits. Odds ratio (OR) with 95% confidence intervals was used to estimate the causal effects. Enrichment analyses were further conducted on genes corresponding to involved single nucleotide polymorphisms.

**Results:**

In forward analysis, IBS potentially increased the connectivity between postcentral/ precentral gyrus and cerebellum (OR = 1.15, *P* = 0.014), and decreased the connectivity between occipital/precuneus and superior frontal lobe (OR = 0.87, *P* = 0.037), as well as the connectivity between cerebellum and subcortical region (OR = 0.86, *P* = 0.017). In reverse analysis, increased neural activity in parietal/postcentral/precuneus region was potentially negatively associated with IBS (OR = 0.93, *P* = 0.036); the connectivity between cerebellum and subcortical region (OR = 1.16, *P* = 0.042) was potentially positively associated with IBS; the connectivity between cerebellum and precentral/frontal/superior motor area (OR = 0.85, *P* = 0.032), calcarine/lingual and paracentral gyrus (OR = 0.88, *P* = 0.031), postcentral/precentral and subcortical region (OR = 0.89, *P* = 0.019) were potentially negatively associated with IBS. The abnormally connected brain networks mainly included subcortical-cerebellum, motor, default mode and attention networks. Enrichment analyses demonstrated that the genes were mainly enriched in cell junctions and long-term potentiation pathways.

**Conclusions:**

Our findings provided suggestive evidence for several causal relationship between IBS and rsfMRI phenotypes, in which, cerebellum, subcortical regions, postcentral and precentral gyrus played important roles. These putative causal relationships warrant further validation in independent, ideally larger-scale datasets.

## Introduction

Irritable bowel syndrome (IBS) is one of the most common disorders of gut-brain interaction, characterized by recurrent abdominal pain associated with altered stool form or frequency.^[1]^ An Internet cross-sectional survey conducted by the Rome Foundation across 26 countries reported an overall IBS prevalence of 4.1% according to Rome IV criteria.^[2]^ IBS has a considerable impact on both individuals’ quality of life and global healthcare costs,^[3]^ underscoring the urgent need for effective prevention and intervention measures. However, the underlying causes of IBS remain largely unknown.

A growing body of evidence implicates the gut-brain axis as a key mediator in the pathogenesis of IBS.^[4]^ The frequent coexistence of IBS with common mental disorders, such as depression and anxiety, has been well established.^[5]^ With the advent and development of brain imaging techniques, these reciprocal relationships could be further visualized. Recently, a series of observational studies reported associations between IBS and distinct brain structural and functional signatures.^[6]^ Brain structural abnormalities cannot directly reflect functional abnormalities, and multiple brain regions cooperate to participate in specific brain function; therefore, it is crucial to explore the causal relationships between IBS and brain functional networks.

Resting-state functional magnetic resonance imaging (rsfMRI) is a promising tool to detect spontaneous functionally active brain regions, which is crucial for uncovering the intrinsic brain functional architecture.^[7]^ The local brain function alterations found in patients with IBS in previous studies were widely distributed in multiple parts of the brain, including the postcentral gyrus, inferior parietal gyrus, cerebellum, middle frontal gyrus, inferior temporal gyrus, precuneus, anterior cingulate cortex, and amygdala, among others.^[8, 9, 10]^ Despite these findings, the results remain inconsistent or even contradictory. Furthermore, most studies are cross-sectional, limiting their ability to establish causal relationships and rendering them susceptible to confounding factors. More importantly, the majority of studies laid heavy emphasis on local brain functional activities, often ignoring functional connectivity between brain regions.^[11]^

Mendelian randomization (MR) is a method that utilizes single nucleotide polymorphisms (SNPs) as instrumental variables (IVs) to infer causal relationships between exposures and outcomes.^[12]^ This method can be regarded as a natural randomized controlled trial as alleles are randomly assigned during gamete formation, making it less prone to reverse causality and confounding factors compared to conventional observational studies.^[13]^ Recent large-scale genetic studies on IBS and brain functional networks provide a pivotal opportunity to explore their causal relationships.^[14,15]^

In this study, we conducted a bidirectional two-sample MR analysis by using publicly available genome-wide association study (GWAS) summary statistics of IBS and brain rsfMRI phenotypes, aiming to explore the potential causal relationships between IBS and brain functional networks.

## Methods

### Study design

**Figure 1:**
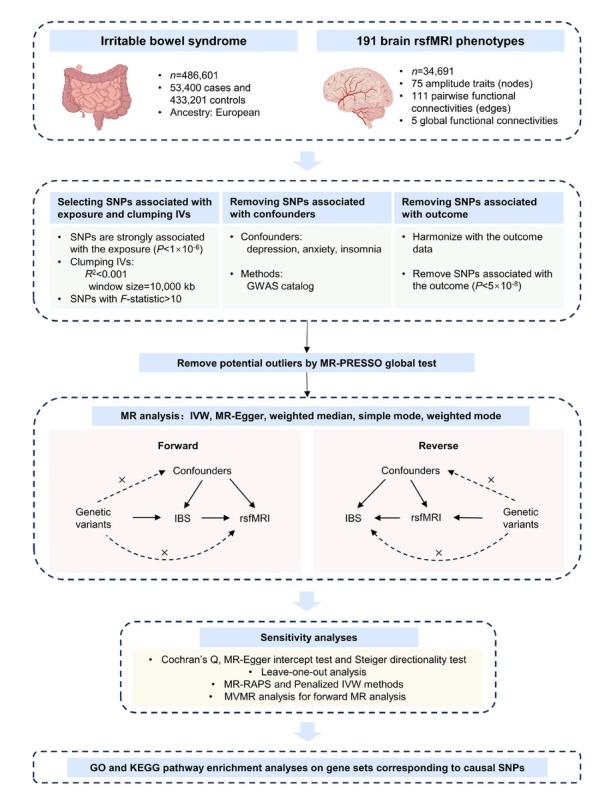
Flow chart of the study. rsfMRI: resting-state functional magnetic resonance imaging; SNP: single nucleotide polymorphism; IV: instrumental variable; MR-PRESSO: Mendelian randomization pleiotropy residual sum and outlier; MR: Mendelian randomization; IVW: inverse variance weighted; GO: Gene Ontology; KEGG: Kyoto Encyclopedia of Genes and Genomes; IBS: irritable bowel syndrome.

This bidirectional MR analysis was performed in accordance with the STROBE-MR Statement guidelines,^[16]^ and the study design overview is shown in Figure 1. The forward MR analysis considered IBS as the exposure and brain functional networks as the outcome, while the reverse MR analysis used brain functional networks as the exposure and IBS as the outcome. The study was based on publicly available data; therefore, no ethical approval or informed consent were needed.

### Data sources

The GWAS summary statistics for IBS were obtained from the IEU database, including 486,601 individuals (53,400 cases and 433,201 controls) of European ancestry, accessible at IEU Open GWAS (https://gwas.mrcieu.ac.uk/datasets/ebi-a-GCST90016564/).^[14]^ Among them, 333,768 individuals (40,548 cases and 293,220 controls) were from the UK Biobank, and 152,833 individuals (12,852 cases and 139,981 controls) were from Bellygenes initiative (an international collaboration).

The summary-level data on brain rsfMRI phenotypes were derived from a genetic study (https://pubmed.ncbi.nlm.nih.gov/35393594/).^[15]^ This study investigated the association between 1777 intrinsic brain activity phenotypes and 9,026,427 common variants in the UK Biobank dataset (34,691 individuals). Considering that the genetic influence on brain function is weaker than its influence on brain structure,^[17]^ the authors filtered the phenotypes that were significantly affected by genetic variants from the 1777 traits. Finally, a total of 191 traits were selected for GWAS, including 75 amplitude traits (nodes) that reflect regional spontaneous neural activities, 111 pairwise functional connectivities (edges) and 5 global functional connectivities, which formed the data source of our study. Further details of the dataset are shown in Supplementary Table S1 and can be found in the original research.

### Selection of instrumental variables

MR analysis relies on three core assumptions: (1) Relevance: The IVs should be strongly associated with the exposure; (2) Independence: The IVs should be independent of confounders-factors that may affect the exposure-outcome causality; (3) Exclusion: The IVs should not be directly related to the outcome, ensuring that any observed causal effect is solely driven by the exposure.^[18]^ To satisfy these assumptions, we filtered the instruments before MR analysis. First, we selected strongly associated SNPs from the GWAS statistics of the exposure. Given the limited number of SNPs, we adjusted the genome-wide significance threshold from the traditional *P* < 5×10^−8^ to *P* < 1×10^−6^, and further relaxed it to *P* < 1×10^−5^ when fewer than four SNPs were available.^[19,20]^ Then, we performed linkage-disequilibrium pruning to select independent SNPs with the following parameters: *R*^2^ < 0.001, window size = 10,000 kb. To mitigate weak instrument bias, we calculated the *F*-statistic for candidate IVs and retained SNPs with *F*-statistic >10.^[21]^ The *F*-statistic was computed as: *F* = [*R*^2^ × (*n* − *k* − 1)]/[(1 − *R*^2^) × *k*], where *R*^2^ represented the extent to which the exposure could be explained by IVs, *n* denoted the sample size of exposure GWAS and *k* was the number of SNPs in IVs. The *R*^2^ was expressed as: *R*^2^ = 2 × *EAF* × (1 − *EAF*) × *β*^2^, where *EAF* was the effect allele frequency, and *β* referred to genetic effect size from exposure GWAS. In the absence of *EAF* in our reverse MR analysis, *R^2^* was calculated using *R*^2^ = *β*^2^/(*β*^2^ + *Se*^2^ × *n*) instead, where *Se* referred to the standard error for effect size of exposure and *n* was the sample size. Next, we used the NHGRI-EBI GWAS catalog (https://www.ebi.ac.uk/gwas/) to remove SNPs significantly associated with confounders.^[22]^ Potential confounders, including depression, anxiety and insomnia, were considered, because these factors have been reported to influence both IBS and brain functional networks.^[23, 24, 25]^ Last, after incorporating the outcome data, SNPs significantly associated with the outcome at a threshold of *P* < 5×10^−8^ were excluded.

### Mendelian randomization pleiotropy residual sum and outlier (MR-PRESSO)

MR-PRESSO method was used to identify potential outlier SNPs with significant horizontal pleiotropy (global test *P* < 0.05), which were removed before MR analysis.^[26]^

### MR analysis

We performed bidirectional MR analysis to explore potential causal relationships between IBS and brain functional networks. Five strategies were utilized: inverse variance weighted (IVW), MR-Egger, weighted median, simple mode, and weighted mode, in which IVW regression with random effect model served as the primary method for causal inference. The IVW method assigns inverse variance as weights and has the highest statistical power when all IVs are valid, but the results may be biased when any of the SNPs has horizontal pleiotropy.^[27]^ The MR-Egger method introduces an intercept to account for horizontal pleiotropy, offering more robust estimates even in the absence of valid IVs.^[28]^ The weighted median method, akin to IVW but utilizing the median rather than the mean, provides reliable estimates provided that at least 50% of the IVs are valid.^[29]^ The mode-based approaches cluster similar variants and estimate the causal effect based on the cluster with the largest number of SNPs.^[30]^

To ensure the robustness of our findings, the following criteria for inferring causal relationships were established:^[31]^ (1) at least four SNPs were available for IVs, and (2) the causal effect estimates of the other four methods were in the same direction as that of the IVW method. For statistical significance, we adopted a tiered threshold approach: (1) the *P* value of IVW method less than 0.05 was considered to be nominally significant results; (2) the *P* value was further corrected using the Bonferroni method and was set as 1.31×10^−4^ (0.05/191/2, where 191 is the number of rsfMRI phenotypes, 2 represents forward and reverse MR analyses) for significant results. Odds ratio (OR) with 95% confidence intervals (CI) was used to indicate the effect size of causality between exposure and outcome.

### Sensitivity analysis

We performed comprehensive sensitivity analyses to assess the robustness of our MR estimates. First, we conducted several analyses to assess core MR assumptions: (1) Cochran’s Q test to detect the heterogeneity of IVs; (2) MR-Egger intercept test to examine the existence of potential horizontal pleiotropy; (3) Steiger directionality test to validate the presumed direction of causality.

Second, leave-one-out analysis was applied to test whether the causal relationships was obviously driven by a particular SNP.

Third, the additional MR-Robust Adjusted Profile Score (MR-RAPS) and Penalized inverse variance weighted (Penalized IVW) regression were employed to obtain causal estimates robust to weak instruments and potential pleiotropy. MR-RAPS accounts for pervasive pleiotropy by modeling it as a random effect, while Penalized IVW mitigates the influence of outlying genetic variants by applying a statistical penalty.^[32,33]^ Both methods provide more conservative causal estimates when the standard IVW assumptions are violated.

Although we had removed the relevant confounding SNPs in the main MR analysis, we performed multivariable MR (MVMR) analyses for estimated significant associations in the forward MR analysis to assess whether the potential causal effects of IBS on brain functional networks were independent of genetic predispositions to major depression, anxiety disorders, and insomnia. The genetic instruments for these covariates were selected from the following GWAS studies (major depression: https://gwas.mrcieu.ac.uk/datasets/ieu-b-102/; anxiety disorders: https://gwas.mrcieu.ac.uk/datasets/finn-b-KRA_PSY_ANXIETY/; insomnia: https://gwas.mrcieu.ac.uk/datasets/ebi-a-GCST90018869/). The MVMR estimates were derived using the IVW method.

### Enrichment analysis

To further explore potential biological mechanisms linking IBS and brain functional networks, we analyzed the gene set corresponding to SNPs involved in established causal relationships. Gene Ontology (GO) and Kyoto Encyclopedia of Genes and Genomes (KEGG) pathway enrichment analyses were performed on these genes. The analyses were completed using the g:Profiler (https://biit.cs.ut.ee/gprofiler)^[34]^ and OmicShare (https://www.omicshare.com)^[35]^ online platforms.

### Statistical software

All statistical analyses were completed using the TwoSampleMR package (version 0.6.2) in R studio (version 4.4.0). Some of the figures were generated using Chiplot (https://www.chiplot.online/) and OmicShare (https://www.omicshare.com) tools.^[35]^ All figures in this manuscript have been optimized to ensure readability.

## Results

**Figure 2:**
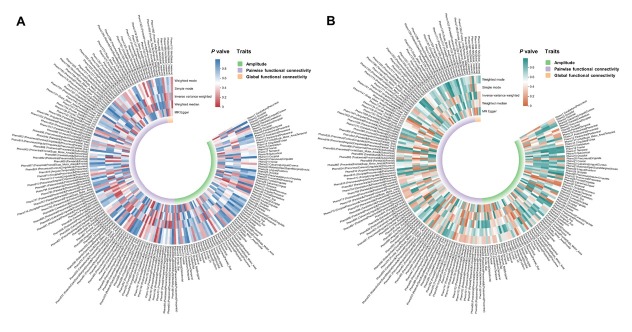
Circular heatmap results of all exposure-outcome pairs in MR analysis. (A) Forward MR analysis of IBS on brain rsfMRI traits. (B) Reverse MR analysis of brain rsfMRI traits on IBS. MR: Mendelian randomization; IBS: irritable bowel syndrome; rsfMRI: resting-state functional magnetic resonance imaging.

The overall results of our MR analysis were presented in Figure 2. In general, the estimated causal relationships between IBS and brain functional connectivities appeared to be stronger than those between IBS and amplitude traits, both in forward and reverse analyses. While no associations survived Bonferroni correction, we found several nominally potential causal associations that may warrant further investigation.

### Forward MR analysis of IBS on brain rsfMRI traits

**Figure 3:**
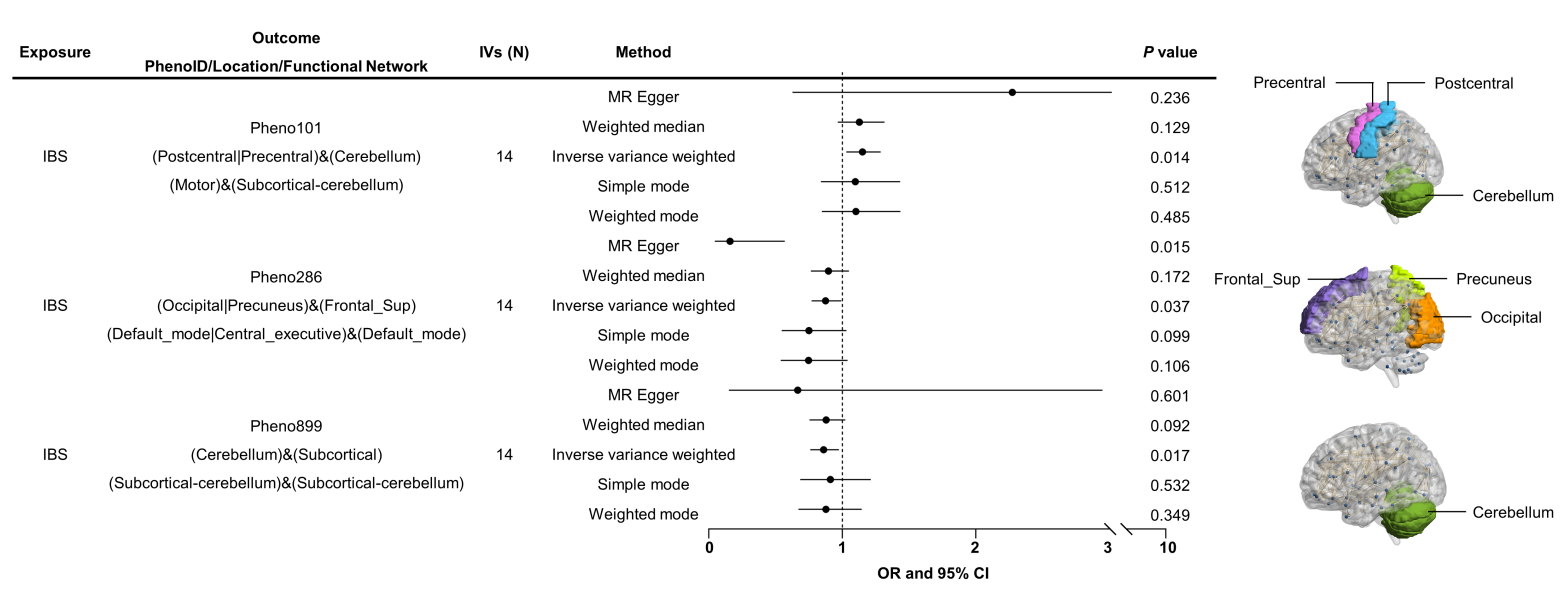
Results of forward MR analysis. Left: Forest plot for MR analysis of IBS on brain rsfMRI traits. OR represented the effect size of 1 standard deviation change in risk of IBS on the mean rsfMRI phenotype: and the error bars represented 95% CI. Right: Pattern diagram showing the brain anatomical region of the corresponding rsfMRI phenotypes. MR: Mendelian randomization; IBS: irritable bowel syndrome; rsfMRI: resting-state functional magnetic resonance imaging; OR: odds ratio; CI: confidence interval.

In forward MR analysis, our results suggested three potential causal relationships of IBS on brain rsfMRI traits (Figure 3, Supplementary Figure S1, Supplementary Table S2 and S3). IBS was potentially associated with an increase in the connectivity between postcentral/precentral gyrus and cerebellum (IVW: OR = 1.15, 95% CI: 1.03 – 1.29, *P* = 0.014), and was potentially associated with a decrease in the connectivity between occipital/precuneus and superior frontal lobe (IVW: OR = 0.87, 95% CI: 0.77–0.99, *P* = 0.037), as well as the connectivity between cerebellum and subcortical region (IVW: OR = 0.86, 95% CI: 0.76–0.97, *P* = 0.017). The risk of IBS might affect connectivity in different brain networks, including subcortical-cerebellum network, default mode network (DMN), motor network and central executive network.

### Reverse MR analysis of brain rsfMRI traits on IBS

**Figure 4:**
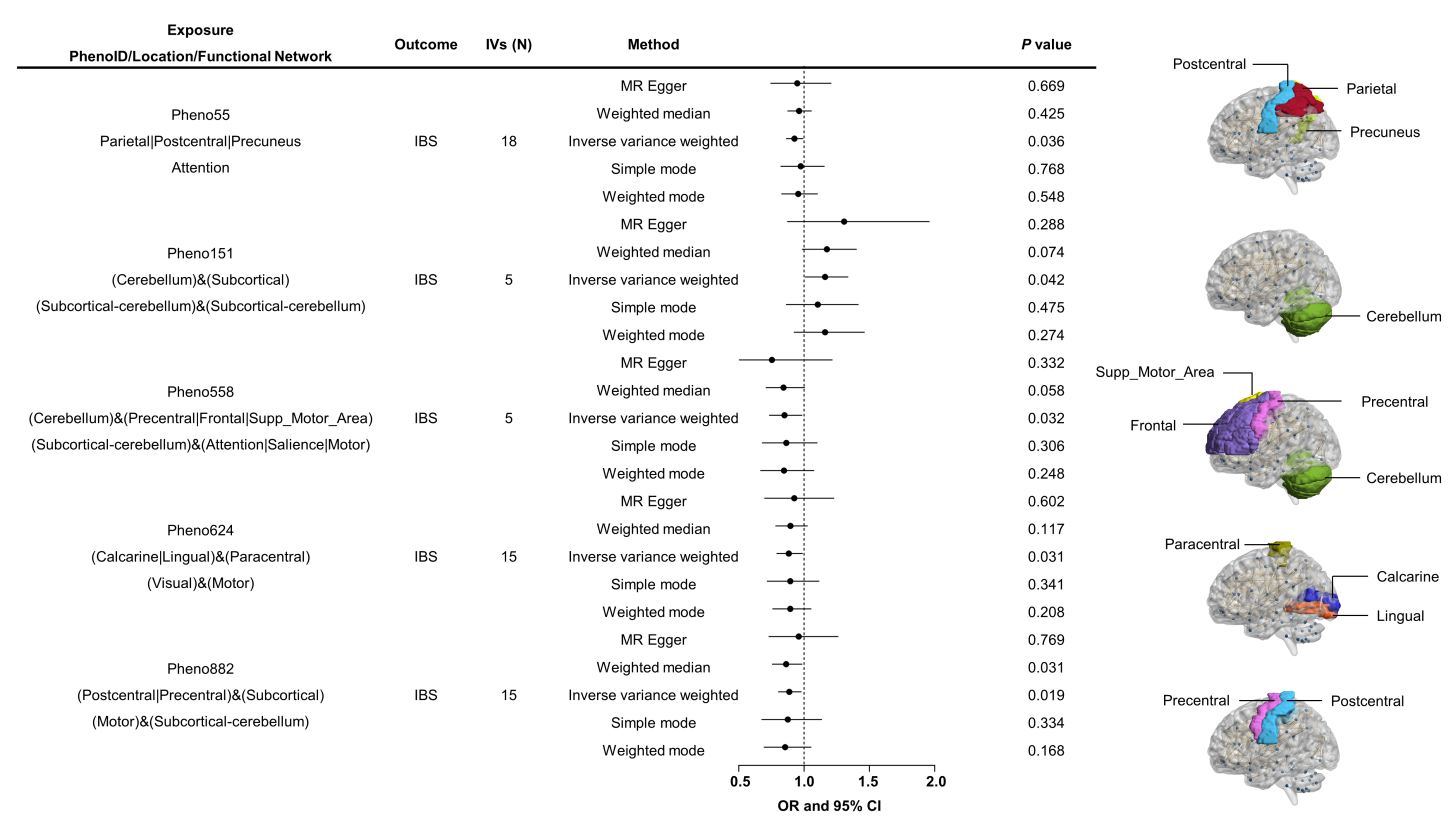
Results of reverse MR analysis. Left: Forest plot for MR analysis of brain rsfMRI traits on IBS. OR represented the effect size of 1 standard deviation change in mean rsfMRI phenotype on the risk of IBS: and the error bars represented 95% CI. Right: Pattern diagram showing the brain anatomical region of the corresponding rsfMRI phenotypes. MR: Mendelian randomization; IBS: irritable bowel syndrome; rsfMRI: resting-state functional magnetic resonance imaging; OR: odds ratio; CI: confidence interval.

In reverse MR analysis, our findings supported five brain rsfMRI traits that might causally affect IBS (Figure 4, Supplementary Figure S1, Supplementary Table S4 and S5). The increase of neural activity in parietal/postcentral/precuneus region was potentially negatively associated with the risk of IBS (IVW: OR = 0.93, 95% CI: 0.86–0.99, *P* = 0.036). The connectivity between cerebellum and subcortical region was potentially positively associated with IBS (IVW: OR = 1.16, 95%CI: 1.01–1.34, *P =* 0.042). The connectivity between cerebellum and precentral/frontal/superior motor area (IVW: OR = 0.85, 95% CI: 0.73–0.99, *P =* 0.032), calcarine/lingual and paracentral gyrus (IVW: OR = 0.88, 95% CI: 0.79–0.99, *P =* 0.031), postcentral/precentral and subcortical region (IVW: OR = 0.89,95% CI: 0.80–0.98, *P =* 0.019) were potentially negatively associated with IBS. The brain networks involved in possible reverse causalities included subcortical-cerebellums networks, motor networks, attention networks, salience network and visual networks.

### Sensitivity analysis

The results of sensitivity analysis for Cochran’s Q, MR-Egger intercept test and Steiger directionality test were shown in Supplementary Table S6. No heterogeneity was observed in all MR results (*P* > 0.05). The MR-Egger intercept differed from zero in the relationship of IBS on Pheno 286 due to the detection of potential horizontal pleiotropy (*P* = 0.022), and horizontal pleiotropy was not detected in the remaining MR estimates (*P* > 0.05). The Steiger directionality test indicated the correct causal direction for all significant findings were “true”. Specifically, the reverse MR analyses yielded strong statistical support (all *P* <1×10^-30^), whereas the *P*-values in the forward MR analyses were non-significant.

Leave-one-out analysis showed that several results were insufficient to support a robust causal inference (Supplementary Figure S2).

The results of MR-RAPS and Penalized IVW methods were presented in Supplementary Table S7. Reassuringly, all potential significant causal associations found in the primary IVW analysis remained statistically significant (*P* < 0.05) using both robust methods. The consistency across these diverse methods strengthened the credibility of our primary causal inferences.

The results of MVMR analyses for estimated significant associations in the forward MR analysis were presented in Supplementary Table S8. The effect of IBS on the connectivity between cerebellum and subcortical region (Pheno 899) retained a nominal significance, while the effects on the other two connectivities were no longer statistically significant.

### Enrichment analysis

**Figure 5:**
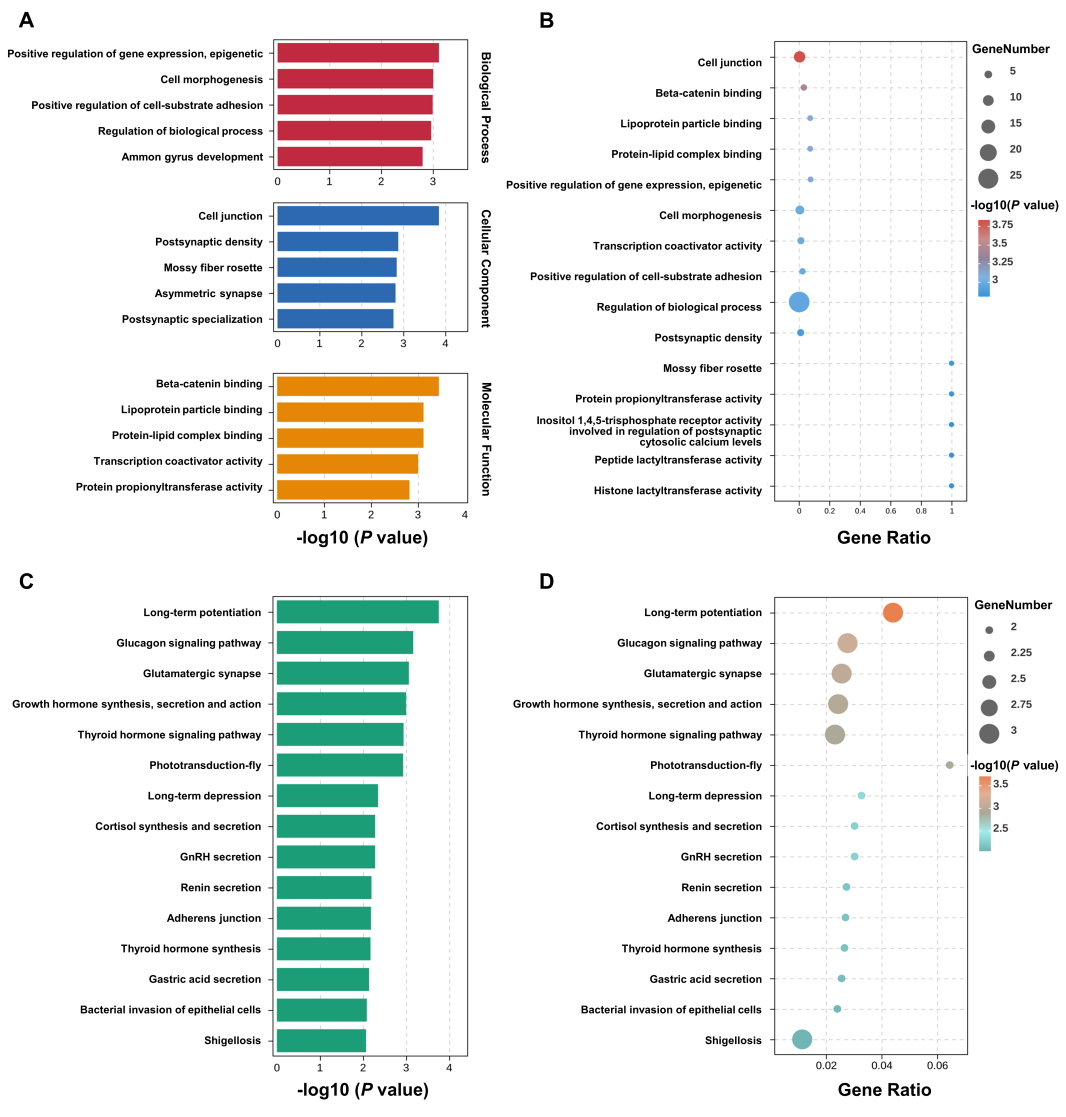
Enrichment analysis of gene set corresponding to causal SNPs. (A) Bar Chart of the top five GO enriched pathways respectively in three categories: biological process: cellular component: and molecular function. (B) Bubble Chart of the top 15 GO enriched pathways. (C) Bar Chart of the top 15 KEGG enriched pathways. (D) Bubble Chart of the top 15 KEGG enriched pathways. SNP: single nucleotide polymorphism; GO: Gene Ontology; KEGG: Kyoto Encyclopedia of Genes and Genomes.

We analyzed SNPs involved in estimated causal relationships and mapped them to the gene database. According to GO enrichment analysis, the genes were mainly enriched in cell junctions, beta-catenin binding and lipoprotein particle binding pathways. As for KEGG enrichment analysis, the genes were primarily involved in the pathway of longterm potentiation (LTP), glucagon signaling, as well as glutamatergic synapse (Figure 5).

## Discussion

**Figure 6:**
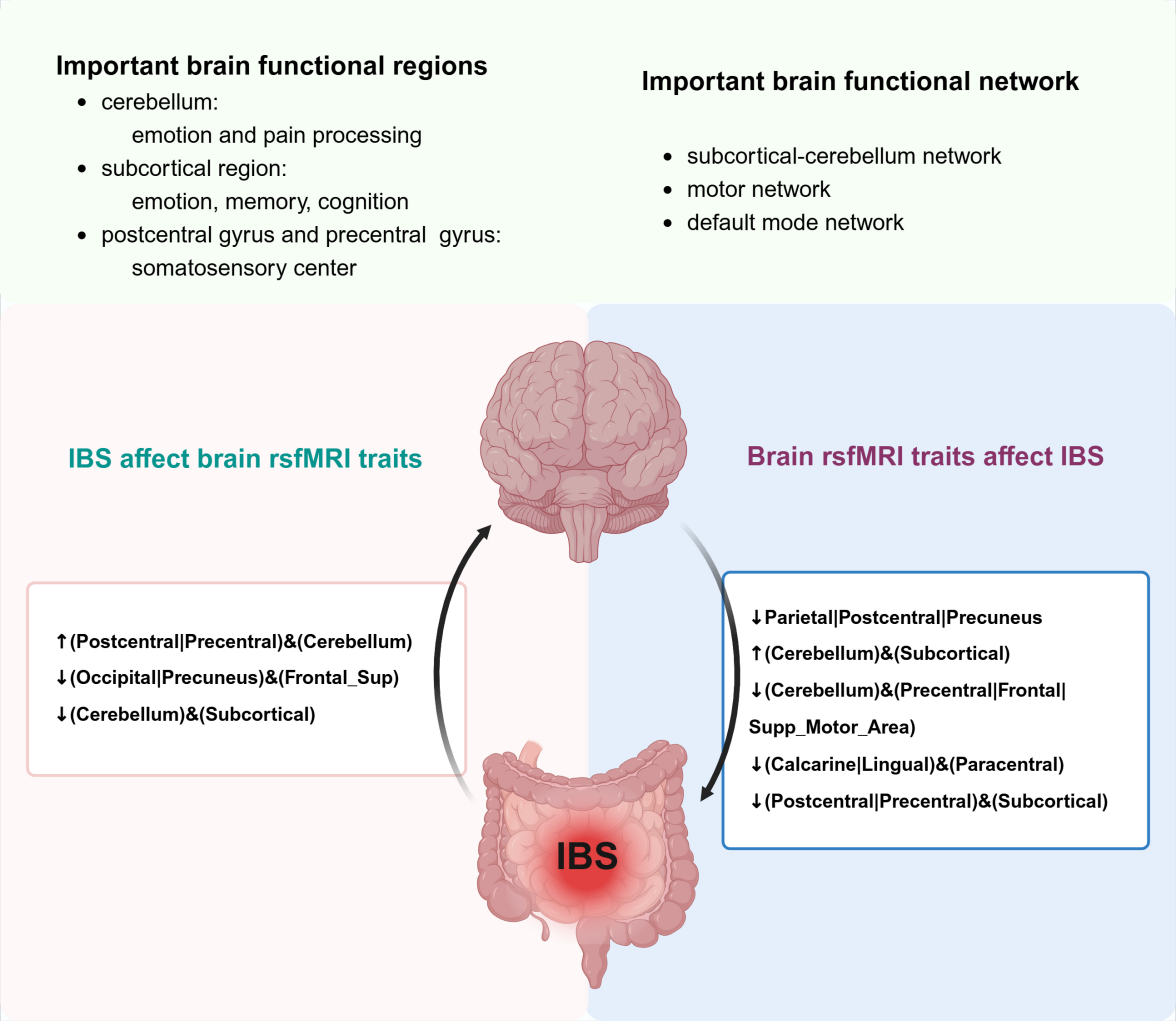
Framework for visualizing the results of MR analysis for IBS and brain functional networks. MR: Mendelian randomization; IBS: irritable bowel syndrome; rsfMRI: resting-state functional magnetic resonance imaging.

In this study, we explored the causal relationships between IBS and 191 brain rsfMRI traits using bidirectional two-sample MR analysis. Our results suggested three potential causal effects of IBS on brain rsfMRI and five potential causal effects of brain rsfMRI on IBS, in which cerebellum, subcortical regions, postcentral and precentral gyrus, as well as subcortical-cerebellum network, motor network, DMN and attention network played critical effects (Figure 6). To the best of our knowledge, this is the first large-sample study to comprehensively investigate the causal relationships between IBS and brain function using genetic data. Our exploratory research not only contributes to a deeper understanding of the pathogenesis of IBS, but also provides new potential targets for the prevention and treatment of IBS.

The bidirectional connection between the gastrointestinal system and the nervous system, known as the “gut-brain axis”, is integral to the development of IBS. This axis regulates visceral sensitivity, gastrointestinal motility, stress response, and cognitive function, involving alterations in brain structure and function as well as increased mental health issues among patients with IBS.^[36]^ Through a comprehensive analysis of the brain in patients with IBS, Liu *et al*. suggested that functional connectivity of brain was more vulnerable to IBS than anatomical connectivity, while structural morphology was the most stable.^[37]^ Our study supported that the relationships between IBS and brain functional connectivities were generally stronger than those between IBS and amplitude traits, also highlighting the significance of functional connectivity.

Our study provided genetic evidence supporting that the dysfunction of functional connectivities between cerebellum and specific brain regions was causally related to IBS. This result aligns with a previous meta-analysis which reported increased activity of cerebellum in patients with IBS.^[9]^ Accumulating evidence suggested that the cerebellum played an important role in emotional, sensory, and cognitive processes.^[38]^ Anatomical and functional connectivity studies have revealed that the cerebellum is extensively interconnected with multiple cortical and subcortical regions which serve various functional domains, enabling it to modulate emotional processes through relevant neural pathways.^[39]^ Furthermore, the cerebellum is a key component of the pain matrix, regulating multidimensional aspects of pain processing.^[40]^ Notably, Claassen *et al*. demonstrated that the cerebellum was more active in visceral pain processing than in somatic pain processing, by comparing its responses to rectal distension and cutaneous heat stimuli.^[41]^ Therefore, the abnormal activity of cerebellum may reflect its modulatory role in mental states such as depression and anxiety, as well as symptom of abdominal pain in patients with IBS.

The subcortical region was another pivotal brain area in our study, with functional connectivities between it and other brain regions showing potential causal relationships with IBS. Subcortical region refers to a group of neural structures located deep within the brain, including the limbic system, diencephalon, pituitary gland, and basal ganglia.^[42]^ These structures are involved in complex activities such as memory, emotion, pleasure, and hormone production.^[43]^ Zhao *et al*. concluded that functional connectivity alterations in patients with IBS were mainly in limbic system (including amygdala, hippocampus and median cingulate gyrus).^[10]^ Similarly, another systematic review highlighted the hyperactivity of amygdala and cingulate cortex in patients with IBS at resting state.^[8]^ The limbic system links emotional and visceral states to cognition and behavior, and its dysfunction is associated with emotion dysregulation, memory impairment and cognitive deficit.^[44]^ Unfortunately, due to the limitations of raw data, we were unable to explore specific subcortical regions causally associated with IBS.

The postcentral gyrus and precentral gyrus also played a crucial role in estimated causal relationships. In structural Magnetic Resonance Imaging (MRI) studies, one of the most consistent findings was increased cortical thickness/ gray matter volume in the postcentral gyrus among patients with IBS.^[45]^ A recent meta-analysis also identified that alterations in local brain activity in patients with IBS included postcentral gyrus.^[9]^ The postcentral gyrus is known as the primary somatosensory center and is also a part of the pain matrix. Hobday *et al*. found that anal (somatic) and rectal (visceral) sensation resulted in similar cortical activation pattern in healthy adults, both including activation of primary somatosensory cortex.^[46]^ Another study also proposed that the cerebral processing of visceral pain involved multiple components, partly similar to the processing of somatic pain,^[47]^ which suggested the possible role of postcentral gyrus in the pathogenesis of IBS. The precentral gyrus is the location of the primary motor cortex. Zeng *et al*. reported that activation of precentral gyrus was associated with proximal stomach distention in patients with functional dyspepsia.^[48]^ However, the precentral gyrus is seldom mentioned in existing literatures on IBS-related brain function, and its role remains to be further explored.

Our study also provided suggestive evidence for functional connectivities of other brain regions, such as the precuneus, frontal, occipital, parietal area, and so on. The relationships between IBS and these brain regions need further research in the future.

The human brain remains active in the absence of explicit tasks or stimuli, resulting in an intrinsic functional architecture. A variety of brain intrinsic networks have been discovered using rsfMRI.^[49]^ There is growing evidence that IBS was associated with dysfunction of functional connectivity in several networks.^[50]^ The putative abnormally connected brain networks associated with IBS in our study mainly included subcortical-cerebellum network, motor network, DMN and attention network.

The subcortical-cerebellum network is an important component of the brain functional networks and it modulates emotional and cognitive functions.^[51]^ Based on a novel method for learning time-varying dynamic Bayesian networks, Sun *et al*. revealed the pivotal role of the subcortical-cerebellum network in emotion processing, and the inter-modular connections among subcortical-cerebellum network and motor, visual association network are more active in the emotional task.^[42]^ Studies also found that patients with depression often shared altered connectivity within subcortical-cerebellum network, despite inter-individual heterogeneity.^[52,53]^

The motor network also emerged as a possible significant network associated with IBS in our study. The brain structures included in motor network are primarily involved in the execution and control of voluntary movements, as well as emotion processing.^[54]^ Grinsvall *et al*. found that gray matter differences in sensorimotor network were associated with visceral sensitivity and might reflect neuroplastic alterations in patients with IBS.^[55]^ Another study revealed abnormal functional connectivity of brain regions in sensorimotor networks among IBS individuals with depressive symptoms.^[56]^

The DMN, a group of widely investigated brain regions in rsfMRI studies, is associated with individuals’ higher-order cognition, emotional process, and ability to adapt to stress.^[57]^ Some researchers have demonstrated its essential role in patients with IBS through studies specifically focused on DMN. For instance, Skrobisz *et al*. discovered that patients with IBS showed abnormal connectivity in DMN.^[58]^ And Qi *et al*. reported that the average functional connectivity of DMN was negatively correlated with symptom severity in patients with IBS.^[59]^

In addition, some studies have indicated that patients with IBS exhibit specific abnormalities in the attention network, and these deficits may underlie symptoms such as anxiety, hypervigilance and visceral hypersensitivity.^[60]^ Dysfunction of the attention network may lead to inappropriate allocation of attention resources to somatic cues, resulting in gastrointestinal discomfort, abdominal pain, and other symptoms.^[9]^

There are currently no MR studies on causal relationships between IBS and brain function, and only two relevant meta-analyses have been published. One of the two studies identified widespread local brain activity alterations in patients with IBS, with the most consistent abnormalities being in right postcentral gyrus, left cerebellum, left calcarine fissure and surrounding cortex, left anterior cingulate and paracingulate gyrus and right insula.^[9]^ In another study, patients with IBS showed increased functional connectivity in left inferior parietal gyrus, left lingual gyrus, right angular gyrus, and right precuneus, as well as decreased functional connectivity in right amygdala, right median cingulate gyrus, and left hippocampus.^[10]^ Despite these findings, there is no consensus on relationships between IBS and brain functional networks. Consistent with previous studies, our MR results also indicated a complex landscape of IBS and brain rsfMRI phenotypes, which reflected the complex pathophysiological mechanisms of IBS. Our exploratory study provided possible evidence for relationships between IBS and brain functional networks, and future research using more rigorous designs, such as prospective cohort studies and detailed genetic analyses, are necessary to confirm our findings.

Our bidirectional MR findings revealed a complex and nonlinear interplay within the gut-brain axis that may underpin IBS chronicity. A particularly noteworthy observation was the bidirectional involvement of the connectivity between the cerebellum and subcortical regions. We found that genetic predisposition to IBS potentially decreased connectivity between the cerebellum and subcortical regions, while genetically proxied decreased connectivity in this same circuit was associated with a lower risk of IBS. This seemingly paradoxical finding may, in fact, reflect a disrupted homeostatic feedback loop. Given the established role of this circuit in pain and emotion processing, its dysregulation from either end of the axis could lead to IBS symptoms like abdominal pain and coexistent mental health issues. Clinically, this bidirectional framework may support a dual-target therapeutic strategy for IBS, combining neuromodulation of specific brain networks with peripherally acting gut-directed therapies. Simultaneously intervening on both the “gut-to-brain” and “brain-to-gut” pathways could disrupt the self-perpetuating cycle of IBS, facilitating more holistic and effective disease management.

Enrichment analyses of genes from the putative causal SNPs suggested cell junctions and long-term potentiation (LTP) as the principal enriched pathways. Since these pathways were identified based on SNPs that had genetic associations with both IBS and brain function, they served as potential molecular bridges connecting IBS and brain function. The cell junctions pathway underscored a mechanism of multi-tiered barrier disruption. The integrity of cell junctions is critical not only for intestinal permeability, a process well-established in IBS pathophysiology,^[61,62]^ but also for the integrity of the blood-brain barrier and the stability of synaptic junctions. ^[63,64]^ Thus, genetic variations affecting cell junctions could concurrently compromise the gut barrier, the blood-brain barrier, and synaptic stability, creating a unified vulnerability that facilitates aberrant gut-brain communication. The LTP, a phenomenon in which brief repetitive stimuli lead to a lasting increase in synaptic transmission strength, is generally considered a cornerstone of synaptic plasticity, learning and memory.^[65]^ Notably, LTP has been found to play a critical role in animal models of neuropathic and inflammatory pain.^[66]^ Enteric synaptic plasticity with LTP contributed to colonic visceral hypersensitivity in IBS rats induced by water avoidance stress.^[67]^ Lv *et al*. reported that visceral hypersensitivity in colitis rats may be attributed to enhanced LTP of nociceptive synaptic transmission in the spinal dorsal horn.^[68]^ This suggests that the chronicity of IBS may be rooted in long-lasting, maladaptive alternations in the brain’s functional architecture, driven by LTP processes. Overall, these enrichment analyses provided suggestive insights, and the precise mechanisms underlying interaction between IBS and brain functional networks remain to be further explored in clinical and animal studies.

Several important considerations should be noted when interpreting our results. First, a potential sample overlap exists between the IBS and brain rsfMRI GWAS datasets, as the IBS GWAS summary statistics incorporate data from the UK Biobank, from which the rsfMRI data were exclusively derived. Sample overlap could, in theory, inflate type I error rates in MR analysis. However, the IBS GWAS data included a large number of individuals from non-UK Biobank sources, substantially diluting the influence of overlap. More critically, the consistent results obtained using MR-RAPS, a method robust to bias from sample overlap, provided evidence that our findings were not merely artifacts of sample overlap. Second, the Steiger directionality test indicated the correct causal direction for all significant associations but lacked statistical power in forward MR analyses. This is likely because IBS (a complex, polygenic syndrome with a diffuse genetic architecture) yielded weaker genetic signals than brain imaging phenotypes (higher heritability and precise measurement), thereby reducing the test’s power to achieve formal significance in forward MR analyses. Crucially, the principal support for causal directionality in our study comes from the bidirectional MR design itself. The fact that the three significant forward MR associations did not appear as significant in the reverse direction suggests the presence of direction-specific causal pathways. Third, we detected potential horizontal pleiotropy in the putative relationship of IBS on Pheno286 (*P =* 0.022). Although we had adjusted for major psychiatric confounders including depression, anxiety and insomnia, residual pleiotropy may also stem from more subtle stress response or other psychiatric traits, as well as from immune or metabolic pathways shared between IBS and brain function, which were not fully captured by our covariate adjustment. Importantly, we further addressed this concern by employing MR-RAPS and Penalized IVW methods, which are specifically designed to provide reliable causal estimates in the presence of pleiotropy. The consistent results obtained from these robust methods, along with the concordant directional estimates across all five MR approaches, strengthened our confidence in the observed association despite the detected pleiotropy. Last, our MVMR analysis revealed that IBS had potential independent causal effects on the cerebellum-subcortical connectivity independent of major psychiatric risks, further highlighting its significant role in our findings. However, the other two putative causal effects of IBS on brain connectivity were not independent of genetic liabilities to depression, anxiety, and insomnia. This suggests that the identified associations likely reflect a shared genetic substrate, whereby pleiotropic factors concurrently increase risk for both affective disorders and IBS.

There are significant strengths for our study. First, we employed MR analysis to explore the relationships between IBS and brain functional networks, thus controlling for the possibility of reverse causality and confounding factors. Second, the original genetics-based study included far more patients than previous observational studies. Third, we first performed MR analysis using rsfMRI data. Compared with structural MRI, rsfMRI can directly reflect the functional activity of specific brain regions and reveal the intrinsic network of human brain.

Despite these strengths, this study had several limitations. First, the study populations were mainly derived from European cohorts, which limited the generalizability of our results across different ethnicities. Second, while all IVs exhibited high *F*-statistic, indicating minimal weak instrument bias, the limited number of SNPs for some analyses could affect the precision of the estimates and the power to detect effects. Third, the IBS GWAS data encompass all clinical subtypes (*e.g*., diarrhea-predominant IBS, constipation-predominant IBS), which are known to have distinct pathophysiological mechanisms and potentially divergent brain network signatures. Future studies with subtype-stratified genetic data are warranted to elucidate potential subtype-specific causal relationships. This could be achieved either through deep phenotyping within existing biobanks (*e.g*., applying Rome criteria in UK Biobank) or *via* international collaborative efforts aimed at collecting sufficient genetic data for each subtype. Last, the potential causal relationships we identified were largely statistically inferred, and more clinical longitudinal studies are needed to validate these results in the future.

## Conclusion

In summary, our MR analysis provided suggestive evidence for several causal relationships between IBS and brain rsfMRI phenotypes. IBS was potentially associated with the functional connectivity between cerebellum, subcortical regions, postcentral and precentral gyrus and other brain regions. The abnormally connected brain networks mainly included subcortical-cerebellum network, motor network, DMN and attention network. Our findings provided new insights into the pathogenesis and potential intervention targets for IBS at the brain functional network level. These putative causal relationships warrant further validation in independent, ideally larger-scale datasets, as well as through more clinical and animal studies.

## Supplementary Material

Supplementary Material Details
