# Supplementary material for: Causal relationships between irritable bowel syndrome and brain functional networks: A Mendelian randomization study
Source: J Transl Int Med. 2026 Jun 13;14(3):423–35. doi: 10.1515/jtim-2026-0047 (PMC13320533; doi:10.1515/jtim-2026-0047)
Supplement: Supplementary file 1 — Supplementary Material Details [file jtim-2026-0047_sm.pdf]

Supplementary materials

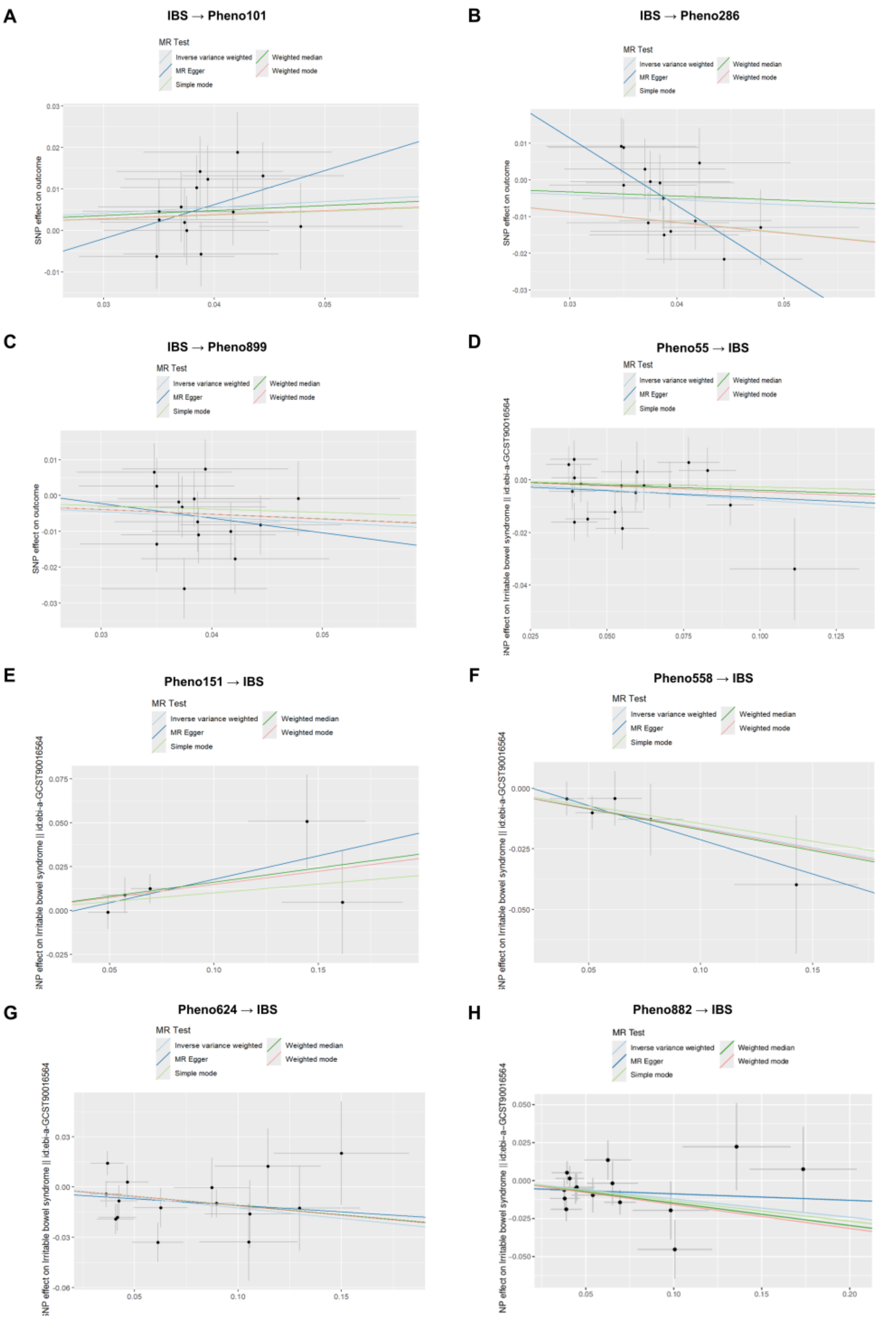

Supplementary Figure S1: Scatter plots of significant results from MR analysis. (A) Result of IBS on Pheno101. (B) Result of IBS on Pheno286. (C) Result of IBS on Pheno899. (D) Result of Pheno55 on IBS. (E) Result of Pheno151 on IBS. (F) Result of Pheno558 on IBS. (G) Result of Pheno624 on IBS. (H) Result of Pheno882 on IBS. MR: Mendelian randomization; IBS: irritable bowel syndrome.

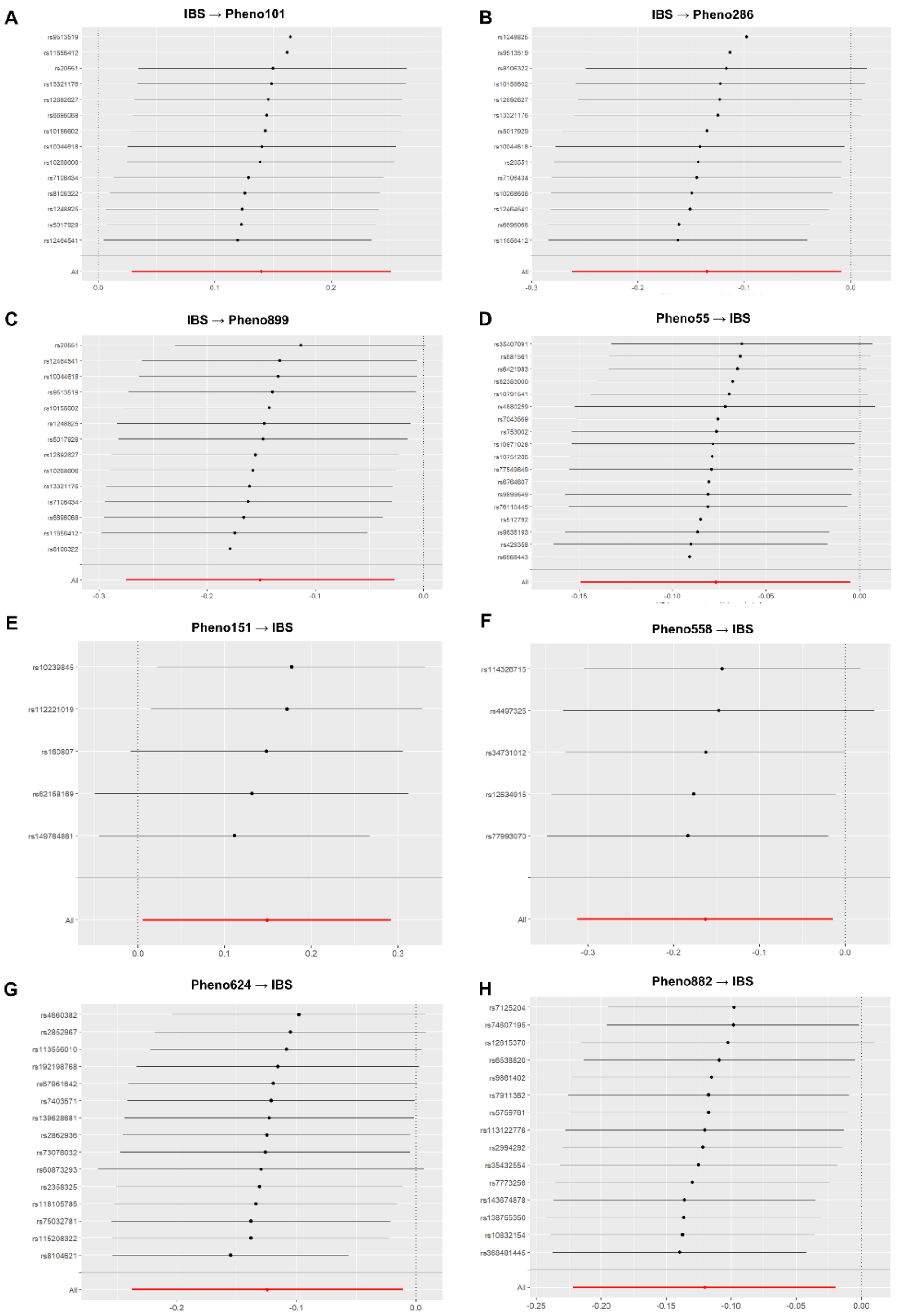

Supplementary Figure S2: Leave-one-out analysis of significant results from MR analysis. (A) Result of IBS on Pheno101. (B) Result of IBS on Pheno286. (C) Result of IBS on Pheno899. (D) Result of Pheno55 on IBS. (E) Result of Pheno151 on IBS. (F) Result of Pheno558 on IBS. (G) Result of Pheno624 on IBS. (H) Result of Pheno882 on IBS. MR: Mendelian randomization; IBS: Irritable bowel syndrome.

Supplementary Table S1. The brain rsfMRI phenotypes list.

**Pheno ID**	**Location**	**Functional Network**
Pheno1	Cerebellum|Precuneus	Default_mode
Pheno2	Occipital	Visual|Attention
Pheno3	Pariental	Attention|Salience|Central_executive
Pheno4	Calcarine|Lingual|Cuneus	Visual
Pheno5	Parietal|Cerebellum	Central_executive|Attention|Default_mode
Pheno7	Precuneus|Occipital	Default_mode|Central_executive
Pheno8	Occipital	Visual
Pheno9	Parietal|Temporal	Default_mode|Central_executive
Pheno10	Postcentral|Precentral	Motor
Pheno11	Postcentral|Precentral	Motor
Pheno12	Paracentral|Postcentral	Motor
Pheno13	Frontal|Supp_Motor_Area|Temporal	Default_mode|Salience
Pheno14	Cingulate|Frontal	Default_mode|Central_executive|Salience
Pheno15	Cerebellum	Subcortical-cerebellum
Pheno16	Frontal	Salience|Central_executive
Pheno17	Temporal	Default_mode|Motor
Pheno18	Subcortical	Subcortical-cerebellum
Pheno19	Occipital	Visual
Pheno20	Precuneus|Cingulate	Default_mode|Central_executive
Pheno21	Frontal	Default_mode|Central_executive
Pheno22	Calcarine|Lingual|Cuneus	Visual
Pheno23	RolandicOper|SupraMarginal|Insula	Salience|Motor
Pheno24	Lingual|Fusiform	Visual
Pheno25	Occipital	Attention|Visual
Pheno26	Precuneus|Angular|Cingulate	Default_mode|Central_executive
Pheno27	Postcentral|Precentral	Motor
Pheno28	Frontal|Cingulate	Default_mode|Limbic
Pheno29	Calcarine|Lingual	Visual
Pheno30	Temporal	Default_mode
Pheno31	OccipitalPrecuneus	Default_mode|Central_executive
Pheno32	Angular|Temporal	Default_mode|Central_executive
Pheno33	Frontal|Cerebellum	Central_executive|Default_mode
Pheno34	Frontal_Sup	Default_mode
Pheno35	Cuneus|Occipital	Visual
Pheno36	Insula|Cingulate	Salience|Default_mode
Pheno37	Occipital	Visual
Pheno38	Cerebellum	Subcortical-cerebellum
Pheno39	Parietal	Attention|Central_executive|Salience
Pheno40	Precuneus|Cuneus|Cingulate	Default_mode|Central_executive
Pheno41	Postcentral|Precentral	Motor|Attention

Pheno42	Frontal_Sup	Default_mode|Central_executive
Pheno43	Temporal_Sup	Default_mode|Motor
Pheno44	Cerebellum	Subcortical-cerebellum
Pheno45	Parietal	Central_executive|Attention
Pheno46	Frontal|Precentral	Central_executive
Pheno47	Parietal|Frontal	Attention|Central_executive
Pheno48	Precentral|Frontal|Supp_Motor_Area	Attention|Salience|Motor
Pheno49	Supp_Motor_Area|Frontal	Salience|Default_mode
Pheno50	Parietal	Central_executive|Salience
Pheno51	Paracentral	Motor
Pheno52	Frontal	Central_executive
Pheno53	Postcentral|Precentral	Motor|Attention
Pheno54	Temporal|Frontal|Supp_Motor_Area	Default_mode|Salience
Pheno55	Parietal|Postcentral|Precuneus	Attention
Pheno56	Postcentral|Precentral	Motor
Pheno57	Precuneus	Default_mode|Central_executive
Pheno58	Frontal	Central_executive|Salience
Pheno59	Subcortical	Subcortical-cerebellum
Pheno60	Precuneus|Parietal_Sup	Attention|Central_executive
Pheno61	Frontal	Central_executive
Pheno62	Temporal|Occipital	Attention|Visual
Pheno63	Temporal|Occipital	Attention|Visual
Pheno64	Temporal|Fusiform	Limbic|Default_mode
Pheno65	Frontal_Inf	Default_mode|Central_executive
Pheno66	Frontal	Salience|Default_mode
Pheno67	Temporal	Central_executive|Default_mode
Pheno68	Frontal	Central_executive|Salience|Default_mode
Pheno69	Temporal	Default_mode|Central_executive
Pheno70	Temporal_Mid|Angular	Default_mode
Pheno71	Orbitofrontal	Limbic
Pheno72	Cerebellum|Temporal	Subcortical-cerebellum
Pheno73	Cerebellum|Temporal	Subcortical-cerebellum
Pheno74	Frontal	Limbic
Pheno75	Temporal|Orbitofrontal	Limbic
Pheno76	Frontal_Sup	Limbic|Default_mode
Pheno101	(Postcentral|Precentral)&(Cerebellum)	(Motor)&(Subcortical-cerebellum)
Pheno1013	(Cerebellum)&(Temporal|Occipital)	(Subcortical-cerebellum)&(Attention|Visual)
Pheno102	(Postcentral|Precentral)&(Cerebellum)	(Motor)&(Subcortical-cerebellum)
Pheno1020	(Paracentral)&(Temporal|Occipital)	(Motor)&(Attention|Visual)
Pheno1022	(Postcentral|Precentral)&(Temporal|Occipital)	(Motor|Attention)&(Attention|Visual)
Pheno103	(Paracentral|Postcentral)&(Cerebellum)	(Motor)&(Subcortical-cerebellum)

Pheno1041	(Angular|Temporal)&(Temporal|Occipital)	(Default_mode|Central_executive)&(Attention|Visual)
Pheno1059	(Parietal)&(Temporal|Occipital)	(Central_executive|Salience)&(Attention|Visual)
Pheno1122	(Temporal)&(Frontal_Inf)	(Default_mode)&(Default_mode|Central_executive)
Pheno1126	(Frontal_Sup)&(Frontal_Inf)	(Default_mode)&(Default_mode|Central_executive)
Pheno1134	(Frontal_Sup)&(Frontal_Inf)	(Default_mode|Central_executive)&(Default_mode|Central_executive)
Pheno1137	(Parietal)&(Frontal_Inf)	(Central_executive|Attention)&(Default_mode|Central_executive)
Pheno1141	(Supp_Motor_Area|Frontal)&(Frontal_Inf)	(Salience|Default_mode)&(Default_mode|Central_executive)
Pheno1142	(Parietal)&(Frontal_Inf)	(Central_executive|Salience)&(Default_mode|Central_executive)
Pheno1161	(Precuneus|Angular|Cingulate)&(Frontal)	(Default_mode|Central_executive)&(Salience|Default_mode)
Pheno1167	(Angular|Temporal)&(Frontal)	(Default_mode|Central_executive)&(Salience|Default_mode)
Pheno1171	(Insula|Cingulate)&(Frontal)	(Salience|Default_mode)&(Salience|Default_mode)
Pheno1175	(Precuneus|Cuneus|Cingulate)&(Frontal)	(Default_mode|Central_executive)&(Salience|Default_mode)
Pheno1183	(Precentral|Frontal|Supp_Motor_Area)&(Frontal)	(Attention|Salience|Motor)&(Salience|Default_mode)
Pheno1184	(Supp_Motor_Area|Frontal)&(Frontal)	(Salience|Default_mode)&(Salience|Default_mode)
Pheno1189	(Temporal|Frontal|Supp_Motor_Area)&(Frontal)	(Default_mode|Salience)&(Salience|Default_mode)
Pheno1205	(Precuneus|Angular|Cingulate)&(Temporal)	(Default_mode|Central_executive)&(Central_executive|Default_mode)
Pheno1211	(Angular|Temporal)&(Temporal)	(Default_mode|Central_executive)&(Central_executive|Default_mode)
Pheno1221	(Frontal_Sup)&(Temporal)	(Default_mode|Central_executive)&(Central_executive|Default_mode)
Pheno1225	(Frontal|Precentral)&(Temporal)	(Central_executive)&(Central_executive|Default_mode)
Pheno1250	(Precuneus|Angular|Cingulate)&(Frontal)	(Default_mode|Central_executive)&(Central_executive|Salience|Default_mode)
Pheno1256	(Angular|Temporal)&(Frontal)	(Default_mode|Central_executive)&(Central_executive|Salience|Default_mode)
Pheno1257	(Frontal|Cerebellum)&(Frontal)	(Central_executive|Default_mode)&(Central_executive|Salience|Default_mode)

Pheno1269	(Parietal)&(Frontal)	(Central_executive|Attention)&(Central_executive|Salience|Default_mode)
Pheno1270	(Frontal|Precentral)&(Frontal)	(Central_executive)&(Central_executive|Salience|Default_mode)
Pheno1273	(Supp_Motor_Area|Frontal)&(Frontal)	(Salience|Default_mode)&(Central_executive|Salience|Default_mode)
Pheno1276	(Frontal)&(Frontal)	(Central_executive)&(Central_executive|Salience|Default_mode)
Pheno1293	(RolandicOper|SupraMarginal|Insula)&(Temporal)	(Salience|Motor)&(Default_mode|Central_executive)
Pheno1296	(Precuneus|Angular|Cingulate)&(Temporal)	(Default_mode|Central_executive)&(Default_mode|Central_executive)
Pheno1300	(Temporal)&(Temporal)	(Default_mode)&(Default_mode|Central_executive)
Pheno1301	(OccipitalPrecuneus)&(Temporal)	(Default_mode|Central_executive)&(Default_mode|Central_executive)
Pheno1302	(Angular|Temporal)&(Temporal)	(Default_mode|Central_executive)&(Default_mode|Central_executive)
Pheno1309	(Parietal)&(Temporal)	(Attention|Central_executive|Salience)&(Default_mode|Central_executive)
Pheno1311	(Postcentral|Precentral)&(Temporal)	(Motor|Attention)&(Default_mode|Central_executive)
Pheno1317	(Parietal|Frontal)&(Temporal)	(Attention|Central_executive)&(Default_mode|Central_executive)
Pheno1319	(Supp_Motor_Area|Frontal)&(Temporal)	(Salience|Default_mode)&(Default_mode|Central_executive)
Pheno132	(Paracentral|Postcentral)&(Temporal)	(Motor)&(Default_mode|Motor)
Pheno1322	(Frontal)&(Temporal)	(Central_executive)&(Default_mode|Central_executive)
Pheno1325	(Parietal|Postcentral|Precuneus)&(Temporal)	(Attention)&(Default_mode|Central_executive)
Pheno1328	(Frontal)&(Temporal)	(Central_executive|Salience)&(Default_mode|Central_executive)
Pheno135	(Cerebellum)&(Temporal)	(Subcortical-cerebellum)&(Default_mode|Motor)
Pheno1359	(Frontal_Sup)&(Temporal_Mid|Angular)	(Default_mode|Central_executive)&(Default_mode)
Pheno1382	(Frontal_Inf)&(Temporal_Mid|Angular)	(Default_mode|Central_executive)&(Default_mode)
Pheno146	(Postcentral|Precentral)&(Subcortical)	(Motor)&(Subcortical-cerebellum)
Pheno147	(Postcentral|Precentral)&(Subcortical)	(Motor)&(Subcortical-cerebellum)
Pheno151	(Cerebellum)&(Subcortical)	(Subcortical-cerebellum)&(Subcortical-cerebellum)
Pheno249	(Lingual|Fusiform)&(OccipitalPrecuneus)	(Visual)&(Default_mode|Central_executiv

		e)
Pheno253	(Frontal|Cingulate)&(OccipitalPrecuneus)	(Default_mode|Limbic)&(Default_mode|Central_executive)
Pheno262	(Frontal|Cingulate)&(Angular|Temporal)	(Default_mode|Limbic)&(Default_mode|Central_executive)
Pheno286	(OccipitalPrecuneus)&(Frontal_Sup)	(Default_mode|Central_executive)&(Default_mode)
Pheno288	(Frontal|Cerebellum)&(Frontal_Sup)	(Central_executive|Default_mode)&(Default_mode)
Pheno303	(RolandicOper|SupraMarginal|Insula)&(Insula|Cingulate)	(Salience|Motor)&(Salience|Default_mode)
Pheno389	(Calcarine|Lingual)&(Postcentral|Precentral)	(Visual)&(Motor|Attention)
Pheno405	(Precuneus|Angular|Cingulate)&(Frontal_Sup)	(Default_mode|Central_executive)&(Default_mode|Central_executive)
Pheno447	(Postcentral|Precentral)&(Cerebellum)	(Motor)&(Subcortical-cerebellum)
Pheno449	(Calcarine|Lingual)&(Cerebellum)	(Visual)&(Subcortical-cerebellum)
Pheno460	(Precuneus|Cuneus|Cingulate)&(Cerebellum)	(Default_mode|Central_executive)&(Subcortical-cerebellum)
Pheno461	(Postcentral|Precentral)&(Cerebellum)	(Motor|Attention)&(Subcortical-cerebellum)
Pheno491	(Precuneus|Angular|Cingulate)&(Frontal|Precentral)	(Default_mode|Central_executive)&(Central_executive)
Pheno537	(RolandicOper|SupraMarginal|Insula)&(Precentral|Frontal|Supp_Motor_Area)	(Salience|Motor)&(Attention|Salience|Motor)
Pheno55	Parietal|Postcentral|Precuneus	Attention
Pheno558	(Cerebellum)&(Precentral|Frontal|Supp_Motor_Area)	(Subcortical-cerebellum)&(Attention|Salience|Motor)
Pheno574	(Frontal_Sup)&(Supp_Motor_Area|Frontal)	(Default_mode)&(Salience|Default_mode)
Pheno58	Frontal	Central_executive|Salience
Pheno590	(RolandicOper|SupraMarginal|Insula)&(Parietal)	(Salience|Motor)&(Central_executive|Salience)
Pheno593	(Angular|Cingulate)&(Parietal)	(Default_mode|Central_executive)&(Central_executive|Salience)
Pheno597	(Temporal)&(Parietal)	(Default_mode)&(Central_executive|Salience)
Pheno599	(Angular|Temporal)&(Parietal)	(Default_mode|Central_executive)&(Central_executive|Salience)
Pheno601	(Frontal_Sup)&(Parietal)	(Default_mode)&(Central_executive|Salience)
Pheno606	(Parietal)&(Parietal)	(Attention|Central_executive|Salience)&(Central_executive|Salience)
Pheno609	(Frontal_Sup)&(Parietal)	(Default_mode|Central_executive)&(Centr

		al_executive|Salience)
Pheno621	(Precuneus|Angular|Cingulate)&(Paracentral)	(Default_mode|Central_executive)&(Motor)
Pheno624	(Calcarine|Lingual)&(Paracentral)	(Visual)&(Motor)
Pheno636	(Postcentral|Precentral)&(Paracentral)	(Motor|Attention)&(Motor)
Pheno639	(Cerebellum)&(Paracentral)	(Subcortical-cerebellum)&(Motor)
Pheno65	Frontal_Inf	Default_mode|Central_executive
Pheno66	Frontal	Salience|Default_mode
Pheno674	(Parietal)&(Frontal)	(Central_executive|Salience)&(Central_executive)
Pheno681	(Postcentral|Precentral)&(Postcentral|Precentral)	(Motor)&(Motor|Attention)
Pheno683	(Calcarine|Lingual)&(Postcentral|Precentral)	(Visual)&(Motor|Attention)
Pheno695	(Postcentral|Precentral)&(Postcentral|Precentral)	(Motor|Attention)&(Motor|Attention)
Pheno698	(Cerebellum)&(Postcentral|Precentral)	(Subcortical-cerebellum)&(Motor|Attention)
Pheno705	(Paracentral)&(Postcentral|Precentral)	(Motor)&(Motor|Attention)
Pheno716	(OccipitalPrecuneus)&(Temporal|Frontal|Supp_Motor_Area)	(Default_mode|Central_executive)&(Default_mode|Salience)
Pheno767	(Parietal)&(Parietal|Postcentral|Precuneus)	(Central_executive|Salience)&(Attention)
Pheno777	(Postcentral|Precentral)&(Postcentral|Precentral)	(Motor)&(Motor)
Pheno789	(Parietal)&(Postcentral|Precentral)	(Attention|Central_executive|Salience)&(Motor)
Pheno794	(Cerebellum)&(Postcentral|Precentral)	(Subcortical-cerebellum)&(Motor)
Pheno801	(Paracentral)&(Postcentral|Precentral)	(Motor)&(Motor)
Pheno810	(Precuneus|Angular|Cingulate)&(Precuneus)	(Default_mode|Central_executive)&(Default_mode|Central_executive)
Pheno812	(Frontal|Cingulate)&(Precuneus)	(Default_mode|Limbic)&(Default_mode|Central_executive)
Pheno815	(OccipitalPrecuneus)&(Precuneus)	(Default_mode|Central_executive)&(Default_mode|Central_executive)
Pheno816	(Angular|Temporal)&(Precuneus)	(Default_mode|Central_executive)&(Default_mode|Central_executive)
Pheno824	(Precuneus|Cuneus|Cingulate)&(Precuneus)	(Default_mode|Central_executive)&(Default_mode|Central_executive)
Pheno867	(Precentral|Frontal|Supp_Motor_Area)&(Frontal)	(Attention|Salience|Motor)&(Central_executive|Salience)
Pheno869	(Parietal)&(Frontal)	(Central_executive|Salience)&(Central_executive|Salience)

Pheno87	(Parietal|Temporal)&(Cingulate|Frontal)	(Default_mode|Central_executive)&(Default_mode|Central_executive|Salience)
Pheno882	(Postcentral|Precentral)&(Subcortical)	(Motor)&(Subcortical-cerebellum)
Pheno899	(Cerebellum)&(Subcortical)	(Subcortical-cerebellum)&(Subcortical-cerebellum)
Pheno903	(Precentral|Frontal|Supp_Motor_Area)&(Subcortical)	(Attention|Salience|Motor)&(Subcortical-cerebellum)
Pheno908	(Postcentral|Precentral)&(Subcortical)	(Motor|Attention)&(Subcortical-cerebellum)
Pheno918	(Precuneus|Angular|Cingulate)&(Precuneus|Parietal_Sup)	(Default_mode|Central_executive)&(Attention|Central_executive)
Pheno932	(Precuneus|Cuneus|Cingulate)&(Precuneus|Parietal_Sup)	(Default_mode|Central_executive)&(Attention|Central_executive)
Pheno942	(Parietal)&(Precuneus|Parietal_Sup)	(Central_executive|Salience)&(Attention|Central_executive)
Pheno956	(Precuneus|Angular|Cingulate)&(Frontal)	(Default_mode|Central_executive)&(Central_executive)
Pheno965	(Cuneus|Occipital)&(Frontal)	(Visual)&(Central_executive)
Pheno1696	NA(Global_measure)	Motor|Subcortical-cerebellum
Pheno1697	NA(Global_measure)	Triple_netwoks(Default_mode)
Pheno1698	NA(Global_measure)	Triple_netwoks(Default_mode)
Pheno1699	NA(Global_measure)	Triple_netwoks(Default_mode)
Pheno1701	NA(Global_measure)	Triple_netwoks(Default_mode)

Supplementary Table S2. Information of instrumental variables for significant exposure-outcome pairs in forward Mendelian randomization analysis.

Exposure	Outcome	SNP	Effect_allele	Other_allele	Beta.exposure	Beta.outcome	Se.exposure	Se.outcome	Pval.exposure	Pval.outcome	Samplesize.exposure	Samplesize.outcome	R^2	F
IBS	Pheno101	rs10044618	T	C	0.035	0.00462879	0.0069	0.00773871	4.12E-07	0.54975	486601	32755	0.000601	292.64015
		rs10156602	G	A	-0.0417	-0.00444988	0.0071	0.00801962	4.36E-09	0.578981	486601	32522	0.0008079	393.45413
		rs10268606	C	T	-0.037	-0.0056263	0.0075	0.0083931	8.36E-07	0.502636	486601	33071	0.0005706	277.79018
		rs1036958	A	T	-0.0379	-0.0146119	0.0068	0.00765007	3.00E-08	0.0561279	486601	33099	0.0007175	349.38833
		rs11656412	A	G	0.0348	-0.00626904	0.007	0.00780313	7.78E-07	0.421743	486601	33184	0.0005764	280.64989
		rs12464541	C	T	0.0421	0.0188504	0.0085	0.00956288	7.27E-07	0.0487008	486601	33443	0.000567	276.03793
		rs1248825	C	A	-0.0444	-0.0131131	0.0073	0.00817338	1.20E-09	0.108632	486601	33043	0.0008653	421.44208
		rs12692627	A	G	-0.0373	-0.00189443	0.0076	0.00845599	8.31E-07	0.822731	486601	33042	0.0005656	275.36661
		rs13321176	G	T	-0.0478	-0.000976731	0.0092	0.010426	1.80E-07	0.925362	486601	32535	0.0006454	314.24644
		rs150079703	G	C	-0.0417	-0.00153223	0.0082	0.00800791	4.21E-07	0.848259	486601	31053	0.0008349	406.58906
		rs20551	G	A	0.0375	-4.80E-06	0.0075	0.00837584	6.16E-07	0.999542	486601	33443	0.0005732	279.09707

		rs2736155	C	G	0.0441	0.00301135	0.007	0.00759745	3.88E-10	0.691837	486601	33443	0.0009701	472.52127
		rs5017929	G	A	-0.0387	-0.0142298	0.0075	0.00835752	2.12E-07	0.0886361	486601	32638	0.0006279	305.75137
		rs541003	C	G	0.0349	0.00171678	0.0068	0.00762476	3.20E-07	0.821856	486601	33430	0.0006076	295.82101
		rs6696068	T	G	-0.035	-0.00256533	0.007	0.00785435	5.80E-07	0.743961	486601	33040	0.0005824	283.54477
		rs7106434	C	T	-0.0384	-0.0103245	0.0069	0.00778168	3.19E-08	0.184583	486601	33311	0.0007131	347.25402
		rs8106322	G	A	0.0394	0.012323	0.0075	0.0081309	1.45E-07	0.129625	486601	32000	0.00071	345.73066
		rs9513519	G	A	-0.0388	0.00572407	0.007	0.00784279	3.09E-08	0.465481	486601	33314	0.0007116	346.53228
		rs996762	G	C	-0.0499	0.0178543	0.0094	0.0105477	1.12E-07	0.0905107	486601	33289	0.0006612	321.93197
		rs10044618	T	C	0.035	-0.00144696	0.0069	0.00765124	4.12E-07	0.850003	486601	32756	0.000601	292.64015
		rs10156602	G	A	-0.0417	0.0111615	0.0071	0.00792914	4.36E-09	0.159235	486601	32523	0.0008079	393.45413
		rs10268606	C	T	-0.037	-0.00291599	0.0075	0.00829847	8.36E-07	0.725296	486601	33072	0.0005706	277.79018
IBS	Pheno286	rs1036958	A	T	-0.0379	-0.00791322	0.0068	0.00756403	3.00E-08	0.295485	486601	33100	0.0007175	349.38833
		rs11656412	A	G	0.0348	0.00919796	0.007	0.00771526	7.78E-07	0.233192	486601	33185	0.0005764	280.64989
		rs12464541	C	T	0.0421	0.0045987	0.0085	0.00945559	7.27E-07	0.626721	486601	33444	0.000567	276.03793

		rs1248825	C	A	-0.0444	0.0216677	0.0073	0.00808107	1.20E-09	0.00733376	486601	33044	0.0008653	421.44208
		rs12692627	A	G	-0.0373	0.0117359	0.0076	0.00836061	8.31E-07	0.160404	486601	33043	0.0005656	275.36661
		rs13321176	G	T	-0.0478	0.012978	0.0092	0.0103085	1.80E-07	0.208042	486601	32536	0.0006454	314.24644
		rs150079703	G	C	-0.0417	0.00769609	0.0082	0.00791729	4.21E-07	0.33102	486601	31054	0.0008349	406.58906
		rs20551	G	A	0.0375	-0.000471048	0.0075	0.00828136	6.16E-07	0.95464	486601	33444	0.0005732	279.09707
		rs2736155	C	G	0.0441	0.00040987	0.007	0.00751185	3.88E-10	0.956487	486601	33444	0.0009701	472.52127
		rs5017929	G	A	-0.0387	0.00514358	0.0075	0.00826365	2.12E-07	0.533656	486601	32639	0.0006279	305.75137
		rs541003	C	G	0.0349	-0.00665667	0.0068	0.00753884	3.20E-07	0.377245	486601	33431	0.0006076	295.82101
		rs6696068	T	G	-0.035	-0.0088334	0.007	0.00776571	5.80E-07	0.255334	486601	33041	0.0005824	283.54477
		rs7106434	C	T	-0.0384	0.000798136	0.0069	0.00769418	3.19E-08	0.917382	486601	33312	0.0007131	347.25402
		rs8106322	G	A	0.0394	-0.0140539	0.0075	0.00803941	1.45E-07	0.0804415	486601	32001	0.00071	345.73066
		rs9513519	G	A	-0.0388	0.0150534	0.007	0.00775445	3.09E-08	0.0522264	486601	33315	0.0007116	346.53228
		rs996762	G	C	-0.0499	0.000414762	0.0094	0.0104293	1.12E-07	0.968277	486601	33290	0.0006612	321.93197
IBS	Pheno899	rs10044618	T	C	0.035	-0.0135375	0.0069	0.00779341	4.12E-07	0.0823805	486601	32752	0.000601	292.64015

		rs10156602	G	A	-0.0417	0.0100162	0.0071	0.00807658	4.36E-09	0.214919	486601	32519	0.0008079	393.45413
		rs10268606	C	T	-0.037	0.00183518	0.0075	0.00845338	8.36E-07	0.828135	486601	33068	0.0005706	277.79018
		rs1036958	A	T	-0.0379	0.0107956	0.0068	0.00770446	3.00E-08	0.161151	486601	33096	0.0007175	349.38833
		rs11656412	A	G	0.0348	0.00656757	0.007	0.0078588	7.78E-07	0.403326	486601	33181	0.0005764	280.64989
		rs12464541	C	T	0.0421	-0.0176881	0.0085	0.00963101	7.27E-07	0.0662723	486601	33440	0.000567	276.03793
		rs1248825	C	A	-0.0444	0.0082154	0.0073	0.00823109	1.20E-09	0.318234	486601	33040	0.0008653	421.44208
		rs12692627	A	G	-0.0373	0.00318858	0.0076	0.0085161	8.31E-07	0.708094	486601	33039	0.0005656	275.36661
		rs13321176	G	T	-0.0478	0.000874316	0.0092	0.0105003	1.80E-07	0.93364	486601	32532	0.0006454	314.24644
		rs150079703	G	C	-0.0417	-0.00725084	0.0082	0.00806473	4.21E-07	0.36861	486601	31050	0.0008349	406.58906
		rs20551	G	A	0.0375	-0.0259495	0.0075	0.0084342	6.16E-07	0.00209307	486601	33440	0.0005732	279.09707
		rs2736155	C	G	0.0441	-0.00752439	0.007	0.00765184	3.88E-10	0.325439	486601	33440	0.0009701	472.52127
		rs5017929	G	A	-0.0387	0.0073007	0.0075	0.00841774	2.12E-07	0.385778	486601	32635	0.0006279	305.75137
		rs541003	C	G	0.0349	0.000383236	0.0068	0.0076792	3.20E-07	0.960198	486601	33427	0.0006076	295.82101
		rs6696068	T	G	-0.035	-0.00257125	0.007	0.0079099	5.80E-07	0.74513	486601	33037	0.0005824	283.54477

		rs7106434	C	T	-0.0384	0.000897881	0.0069	0.00783719	3.19E-08	0.908789	486601	33308	0.0007131	347.25402
		rs8106322	G	A	0.0394	0.00739876	0.0075	0.00818965	1.45E-07	0.366299	486601	31997	0.00071	345.73066
		rs9513519	G	A	-0.0388	0.0110202	0.007	0.00789848	3.09E-08	0.162946	486601	33311	0.0007116	346.53228
		rs996762	G	C	-0.0499	-0.00515682	0.0094	0.0106235	1.12E-07	0.627381	486601	33286	0.0006612	321.93197

Supplementary Table S3. Significant results of forward Mendelian randomization analysis.

**Exposure**	**Outcome**	**Method**	**IVs (N)**	**Beta**	**Se (standard error)**	***P* value**	**Low_CI 95% of beta**	**Up_CI 95% of beta**	**OR**	**Low_CI 95% of OR**	**Up_CI 95% of OR**
IBS	Pheno 101	MR Egger	14	0.820685615	0.65767473	0.235883769	-0.468356856	2.109728086	2.272057061	0.626030082	8.24599878
		Weighted median	14	0.119730057	0.078920512	0.129242244	-0.034954147	0.274414261	1.127192533	0.965649693	1.315759757
		Inverse variance weighted	14	0.140046608	0.056811345	0.013696855	0.028696371	0.251396845	1.150327412	1.029112079	1.285820254
		Simple mode	14	0.092010927	0.136351693	0.511622451	-0.175238391	0.359260244	1.096376802	0.839256926	1.432269493
		Weighted mode	14	0.096787286	0.134559215	0.48469171	-0.166948775	0.360523347	1.101626018	0.846242959	1.43407974
IBS	Pheno 286	MR Egger	14	-1.839294598	0.650258077	0.015216314	-3.113800429	-0.564788766	0.158929496	0.044431774	0.568480216
		Weighted median	14	-0.110938983	0.081175582	0.171733595	-0.270043125	0.048165158	0.894993357	0.763346575	1.049343949
		Inverse variance	14	-0.13515	0.064749353	0.03685	-0.262061447	-0.00824398	0.87358	0.769463741	0.991789904

		weighted		2716		9083		4	2492		
		Simple mode	14	-0.288627782	0.162672002	0.09942224	-0.607464906	0.030209342	0.749291053	0.544730062	1.030670274
		Weighted mode	14	-0.29159882	0.167906502	0.10606467	-0.620695564	0.037497924	0.747068185	0.537570393	1.038209842
IBS	Pheno 899	MR Egger	14	-0.408245909	0.759614821	0.600789506	-1.897090958	1.080599139	0.664815375	0.150004354	2.946444353
		Weighted median	14	-0.13044195	0.077448481	0.092134994	-0.282240972	0.021357072	0.877707442	0.754091948	1.021586766
		Inverse variance weighted	14	-0.151182707	0.063346176	0.017004454	-0.275341213	-0.027024201	0.859690613	0.759312992	0.973337685
		Simple mode	14	-0.093727646	0.146091161	0.532302127	-0.380066322	0.192611029	0.910530715	0.683816056	1.212411109
		Weighted mode	14	-0.132096855	0.136007713	0.349151273	-0.398671972	0.134478263	0.876256122	0.671210841	1.143939793

Supplementary Table S4. Information of instrumental variables for significant exposure-outcome pairs in reserve Mendelian randomization analysis.

Exposure	Outcome	SNP	Effect_allele	Other_allele	Beta.exposure	Beta.outcome	Se.exposure	Se.outcome	Pval.exposure	Pval.outcome	Samplesize.exposure	Samplesize.outcome	R^2	F
Pheno55	IBS	rs10751206	A	G	-0.0414654	0.0014	0.00735926	0.0069	1.76E-08	0.8449	33122	486601	0.0009576	31.745137
		rs10791541	A	G	0.0525435	-0.0122	0.00912721	0.0084	8.57E-09	0.1474	33076	486601	0.001001	33.138714
		rs10971028	C	T	-0.0546937	0.0022	0.010348	0.0096	1.25E-07	0.8219	33079	486601	0.0008438	27.934153
		rs1255	G	C	-0.04119	0.006	0.0079	0.0074	2.29E-0	0.4209	33138	486601	0.000	26.76

		3327			07		6112		7				8072	8485
		rs2274224	C	G	0.0977184	0.0136	0.00732953	0.0069	1.50E-40	0.0477298	33431	486601	0.0052887	177.73561
		rs2276068	C	G	0.0535393	0.0178	0.00729968	0.0068	2.23E-13	0.00924507	33346	486601	0.0016106	53.791256
		rs2431818	T	A	-0.0419678	-6.00E-04	0.00836924	0.0081	5.32E-07	0.9391	31966	486601	0.000786	25.143932
		rs2760748	A	T	-0.0628183	0.023	0.0123049	0.0115	3.31E-07	0.0454695	33250	486601	0.0007832	26.060939
		rs35407091	T	C	-0.0550271	0.0184	0.00874809	0.0082	3.17E-10	0.0240099	33127	486601	0.001193	39.564032
		rs3756187	G	C	0.0382015	0.008	0.00751144	0.007	3.66E-07	0.2572	33431	486601	0.0007731	25.863568
		rs429358	C	T	-0.0766448	-0.0067	0.0101783	0.0095	5.07E-14	0.4831	33431	486601	0.0016933	56.700764
		rs4880259	C	T	0.0903218	-0.0095	0.00762478	0.0079	2.26E-32	0.2301	30571	486601	0.0045691	140.31436
		rs512792	T	C	-0.0374236	-0.0059	0.00732161	0.007	3.20E-07	0.3953	33030	486601	0.0007904	26.12472
		rs55766546	G	C	0.108143	0.0284	0.0214804	0.022	4.79E-07	0.1965	33367	486601	0.000759	25.344636
		rs62383000	A	G	-0.111287	0.0339	0.0212006	0.0193	1.53E-07	0.07942	32669	486601	0.0008427	27.552815
		rs6421983	T	C	-0.0392986	0.016	0.00753778	0.0072	1.85E-07	0.0267399	31696	486601	0.0008568	27.179398
		rs6568443	T	C	-0.0828477	-0.0036	0.00935715	0.0087	8.45E-19	0.675199	33413	486601	0.0023407	78.387666
		rs6764	T	C	0.03932	8.00E-0	0.0074	0.0071	1.40E-0	0.9129	31733	486601	0.000	27.71

		607			41	4	6944		7				8727	4952
		rs681561	C	A	0.0436609	-0.0149	0.00731238	0.0068	2.36E-09	0.0290898	32922	486601	0.0010817	35.648518
		rs7043569	A	G	0.0385682	-0.0043	0.00763991	0.0071	4.46E-07	0.5464	31870	486601	0.000799	25.483261
		rs753002	G	A	0.0593598	-0.0048	0.00733214	0.0069	5.69E-16	0.4809	33258	486601	0.0019669	65.538613
		rs76110445	C	T	-0.0597993	-0.0031	0.0116263	0.0116	2.70E-07	0.7887	33431	486601	0.0007907	26.453502
		rs77549649	A	C	-0.0620207	0.0019	0.0102417	0.0096	1.40E-09	0.8465	32036	486601	0.0011434	36.669257
		rs9635193	T	C	-0.0392002	-0.0079	0.00779992	0.0072	5.02E-07	0.2707	31284	486601	0.0008067	25.256229
		rs9899649	C	T	-0.0704559	0.0019	0.00909119	0.0088	9.20E-15	0.8338	33348	486601	0.0017978	60.057496
Pheno151	IBS	rs10239845	G	A	-0.0492045	0.001	0.00966045	0.0095	3.52E-07	0.9122	33432	486601	0.0007754	25.941134
		rs112221019	A	G	-0.161626	-0.0047	0.0290014	0.0295	2.50E-08	0.8725	33432	486601	0.0009282	31.056931
		rs149764861	C	T	0.144541	0.0507	0.0281779	0.0266	2.90E-07	0.0565497	32945	486601	0.000798	26.311069
		rs160807	T	C	0.0573915	0.0087	0.0113307	0.0103	4.08E-07	0.4003	33383	486601	0.0007679	25.654033
		rs62158169	T	C	-0.0694398	-0.0124	0.00925679	0.0083	6.31E-14	0.1385	33061	486601	0.0016992	56.269082
Pheno558	IBS	rs114326715	A	G	0.142806	-0.0398	0.0278142	0.0285	2.83E-07	0.1618	33320	486601	0.0007905	26.359289
		rs1263	T	C	0.04027	-0.0043	0.0076	0.007	1.64E-0	0.5387	33081	486601	0.000	27.42

		4915			17		9034		7				8283	0956
		rs34731012	T	C	0.0777826	-0.0129	0.0146413	0.0148	1.08E-07	0.3839	32641	486601	0.0008639	28.22143
		rs4497325	G	A	-0.0516914	0.0101	0.00754794	0.0069	7.47E-12	0.1402	33298	486601	0.0014065	46.897925
		rs77993070	T	C	0.0617122	-0.0042	0.0121018	0.0112	3.41E-07	0.704399	33420	486601	0.0007775	26.002561
Pheno624	IBS	rs113556010	T	C	0.0409652	-0.019	0.00908526	0.009	6.51E-06	0.03508	33163	486601	0.0006127	20.329619
		rs115208322	A	C	-0.149948	-0.0201	0.0326487	0.0308	4.37E-06	0.513701	32969	486601	0.0006394	21.092265
		rs118105785	G	A	0.046677	0.0029	0.0103122	0.0099	6.00E-06	0.770801	33212	486601	0.0006165	20.486938
		rs139628681	G	A	0.105718	-0.0161	0.0217595	0.0202	1.18E-06	0.4249	32942	486601	0.000716	23.603352
		rs192198768	G	A	0.105179	-0.0328	0.0237442	0.023	9.44E-06	0.1535	32768	486601	0.0005985	19.62079
		rs2358325	C	T	0.0874309	-4.00E-04	0.0184358	0.0178	2.11E-06	0.9836	33424	486601	0.0006724	22.489507
		rs2852967	A	C	0.0419251	-0.0181	0.00896776	0.008	2.94E-06	0.0229901	33338	486601	0.0006552	21.855171
		rs2862936	G	A	-0.0363562	0.0042	0.00767777	0.0078	2.19E-06	0.5854	33295	486601	0.000673	22.421295
		rs2958514	T	A	0.0342204	-0.0034	0.00773931	0.0069	9.80E-06	0.6182	32871	486601	0.0005944	19.549662
		rs4660382	C	T	0.0613209	-0.033	0.0123561	0.0116	6.95E-07	0.00449904	33401	486601	0.0007368	24.627947
		rs6087	T	G	0.08966	-0.0098	0.0092	0.0082	2.44E-2	0.2351	33370	486601	0.002	94.50

		3293			04		2299		2				8241	0007
		rs67961642	G	A	-0.0627265	0.0123	0.0130113	0.0116	1.43E-06	0.2909	33086	486601	0.000702	23.239912
		rs73076032	A	G	-0.129713	0.0126	0.0292329	0.0254	9.11E-06	0.6205	33078	486601	0.0005949	19.687789
		rs7403571	T	C	0.042511	-0.0083	0.00891805	0.0093	1.87E-06	0.3744	33014	486601	0.0006878	22.721475
		rs75032781	A	G	-0.114461	-0.0123	0.0254439	0.0225	6.84E-06	0.583001	33405	486601	0.0006054	20.235862
		rs8104621	C	T	0.037001	0.0142	0.00797788	0.0072	3.52E-06	0.0507996	33155	486601	0.0006484	21.509273
Phen o882	IBS	rs10832154	A	C	-0.0393638	-0.0052	0.00859727	0.0075	4.68E-06	0.4893	32968	486601	0.0006355	20.962668
		rs11186412	C	G	0.0379821	-0.0194	0.00818551	0.0076	3.48E-06	0.0109901	32920	486601	0.0006536	21.529804
		rs113122776	T	C	-0.0441937	0.0054	0.0095318	0.0099	3.54E-06	0.583201	32462	486601	0.0006618	21.49533
		rs12615370	G	T	0.0694871	-0.0143	0.00892156	0.008	6.77E-15	0.0739401	33080	486601	0.0018305	60.659736
		rs138755350	T	C	0.173582	0.0075	0.0303672	0.0279	1.09E-08	0.787601	33329	486601	0.0009794	32.671857
		rs143674878	A	G	0.135715	0.0223	0.0306921	0.0287	9.79E-06	0.4378	33151	486601	0.0005895	19.551329
		rs2994292	T	C	-0.0449098	0.0044	0.00991529	0.0088	5.92E-06	0.6188	33414	486601	0.0006136	20.513766
		rs35432554	A	G	-0.0651859	0.0018	0.0144775	0.0143	6.71E-06	0.8997	32336	486601	0.0006266	20.271847
		rs3684	T	G	-0.06260	-0.0135	0.0132	0.0129	2.21E-0	0.2937	31501	486601	0.000	22.40

		81445			04		255		6				7107	2807
		rs56217936	A	T	-0.0486452	-9.00E-04	0.0108966	0.0108	8.03E-06	0.9315	30920	486601	0.0006441	19.928283
		rs5759761	A	C	0.054051	-0.0096	0.0118307	0.0114	4.91E-06	0.4015	32416	486601	0.0006435	20.871794
		rs6538820	G	A	0.0380509	-0.0118	0.00841865	0.0077	6.19E-06	0.1263	33434	486601	0.0006106	20.427677
		rs7125204	G	A	0.0389535	-0.0189	0.00880282	0.0077	9.64E-06	0.0140501	32441	486601	0.0006032	19.580445
		rs73225860	T	A	-0.0831739	0.0276	0.0170559	0.0167	1.08E-06	0.0985009	33373	486601	0.0007121	23.779287
		rs74607195	A	G	0.100618	-0.0453	0.0211165	0.0193	1.89E-06	0.0188499	33434	486601	0.0006786	22.702909
		rs7482037	A	T	-0.0396254	-0.0065	0.00776511	0.0069	3.34E-07	0.3452	33157	486601	0.0007848	26.039096
		rs7759673	A	T	-0.0357262	4.00E-04	0.00779458	0.0069	4.57E-06	0.9561	32790	486601	0.0006403	21.006888
		rs7773256	C	A	-0.040889	-0.0014	0.00797858	0.0082	2.98E-07	0.8618	32991	486601	0.0007955	26.262462
		rs7911362	A	G	0.0377812	-0.0062	0.00796511	0.007	2.10E-06	0.381	33113	486601	0.000679	22.497886
		rs9861402	G	A	0.0982965	-0.0196	0.0216393	0.0191	5.56E-06	0.3047	33385	486601	0.0006177	20.633062

Supplementary Table S5. Significant results of reserve Mendelian randomization analysis.

**Exposure**	**Outcome**	**Method**	**IVs (N)**	**Beta**	**Se (standard error)**	***P* value**	**Low_CI 95% of beta**	**Up_CI 95% of beta**	**OR**	**Low_CI 95% of OR**	**Up_CI 95% of OR**

Pheno 55	IBS	MR Egger	18	-0.054169197	0.124535165	0.669393427	-0.298258121	0.189919727	0.947271817	0.742109761	1.209152532
		Weighted median	18	-0.039281454	0.0492035	0.424669293	-0.135720313	0.057157405	0.961480058	0.873086789	1.058822461
		Inverse variance weighted	18	-0.07705973	0.036768473	0.036099056	-0.149125936	-0.004993523	0.925834552	0.861460619	0.995018924
		Simple mode	18	-0.026370709	0.088158652	0.768468474	-0.199161667	0.146420248	0.973973961	0.81941741	1.1576826
		Weighted mode	18	-0.045721112	0.074546	0.547778864	-0.191831272	0.100389047	0.955308349	0.825446133	1.105600965
Pheno 151	IBS	MR Egger	5	0.267527339	0.207517396	0.287741421	-0.139206758	0.674261436	1.306729354	0.870048121	1.962582947
		Weighted median	5	0.161677023	0.090556656	0.074201524	-0.015814022	0.339168068	1.175480527	0.984310363	1.403779255
		Inverse variance weighted	5	0.148817107	0.073046779	0.041621489	0.005645421	0.291988793	1.16046073	1.005661387	1.33908801
		Simple mode	5	0.100032062	0.126941568	0.474760658	-0.148773411	0.348837535	1.105206353	0.861764359	1.417418891
		Weighted mode	5	0.14961245	0.118212135	0.274345242	-0.082083334	0.381308235	1.161384062	0.921195188	1.464198854
Pheno 558	IBS	MR Egger	5	-0.282363649	0.24485071	0.332367902	-0.76227104	0.197543742	0.753999443	0.466605543	1.218406359
		Weighted median	5	-0.171054913	0.090249427	0.058045078	-0.34794379	0.005833964	0.842775293	0.706138567	1.005851015
		Inverse variance weighted	5	-0.163336438	0.076180719	0.032027653	-0.312650648	-0.014022228	0.849305401	0.73150542	0.986075625
		Simple mode	5	-0.145897787	0.124368354	0.305840695	-0.389659761	0.097864187	0.864246036	0.677287275	1.102812999

		Weighted mode	5	-0.166638185	0.123257954	0.247780489	-0.408223774	0.074947403	0.846505834	0.66483009	1.077827459
Pheno 624	IBS	MR Egger	15	-0.077996624	0.146016975	0.602245369	-0.364189895	0.208196648	0.924967549	0.694759251	1.231455308
		Weighted median	15	-0.110745118	0.070559481	0.116524842	-0.249041701	0.027551466	0.895166882	0.779547465	1.027934517
		Inverse variance weighted	15	-0.124404002	0.057831248	0.031463935	-0.237753247	-0.011054756	0.883023026	0.788397206	0.989006124
		Simple mode	15	-0.112093046	0.113721122	0.341017916	-0.334986446	0.110800354	0.893961074	0.715347782	1.117171845
		Weighted mode	15	-0.112093046	0.084876789	0.20779865	-0.278451554	0.054265461	0.893961074	0.756954939	1.05576483
Pheno 882	IBS	MR Egger	15	-0.042148112	0.14027539	0.768572216	-0.317087876	0.232791653	0.958727771	0.728266755	1.262118494
		Weighted median	15	-0.147684876	0.068361353	0.030745102	-0.281673129	-0.013696624	0.86270293	0.754520275	0.986396748
		Inverse variance weighted	15	-0.120714186	0.051504351	0.019090106	-0.221662715	-0.019765658	0.886287236	0.801185547	0.980428402
		Simple mode	15	-0.133441391	0.133460392	0.334348457	-0.395023759	0.128140977	0.875078755	0.673664033	1.136713241
		Weighted mode	15	-0.157359148	0.108226594	0.168001672	-0.369483272	0.054764975	0.854397148	0.691091345	1.056292331

Supplementary Table S6. The results of sensitivity analyses.

**Exposure**	**Outcome**	**Cochran's Q test**		**MR-Egger intercept test**		**Steiger directionality test**	
		**Q value**	***P* value**	**Intercept**	***P* value**	**Correct causal direction**	***P* value**

IBS	Pheno101	9.798	0.71	-0.027	0.319	TRUE	0.215918098
IBS	Pheno286	17.274	0.187	0.067	0.022	TRUE	0.365902134
IBS	Pheno899	15.935	0.253	0.01	0.74	TRUE	0.406540152
Pheno55	IBS	19.956	0.276	-0.001	0.849	TRUE	5.04E-152
Pheno151	IBS	2.464	0.651	-0.009	0.584	TRUE	3.41E-32
Pheno558	IBS	0.773	0.942	0.007	0.644	TRUE	1.88E-30
Pheno624	IBS	21.738	0.084	-0.003	0.733	TRUE	1.41E-72
Pheno882	IBS	16.137	0.305	-0.005	0.556	TRUE	9.72E-69

Supplementary Table S7. The results of MR-RAPS and Penalized IVW methods for significant results.

**Exposure**	**Outcome**	**IVs (N)**	**Beta**	**Se (standard error)**	***P* value**	**OR**	**Low_CI 95% of OR**	**Up_CI 95% of OR**
**MR-RAPS**								
IBS	Pheno101	14	0.146607507	0.061384076	0.016923578	1.1578994	1.026643475	1.305936354
IBS	Pheno286	14	-0.1397903	0.0699915	0.04579839	0.9016284	0.809560357	1.004167019
IBS	Pheno899	14	-0.14388236	0.065704037	0.028534709	0.8659896	0.761349876	0.985011027
Pheno55	IBS	18	-0.07020739	0.035662146	0.04898975	0.9322005	0.86926679	0.99969047
Pheno151	IBS	5	0.150744834	0.076838525	0.049781203	1.1626999	1.000141335	1.351680108
Pheno558	IBS	5	-0.16421336	0.083192801	0.048394353	0.8485609	0.720888172	0.998845193
Pheno624	IBS	15	-0.12545539	0.058497881	0.031983696	0.8820951	0.786540362	0.989258559
Pheno882	IBS	15	-0.11765842	0.051476349	0.022273028	0.8889997	0.803681633	0.983374966
**Penalized IVW**								
IBS	Pheno101	14	0.140046608	0.056811345	0.013696855	1.1503274	1.029112079	1.285820254
IBS	Pheno286	14	-0.13515272	0.064749353	0.036859083	0.8735825	0.769463741	0.991789904
IBS	Pheno899	14	-0.15118271	0.063346176	0.017004454	0.8596906	0.759312992	0.973337685
Pheno55	IBS	18	-0.07705973	0.036768473	0.036099056	0.9258346	0.861460619	0.995018924

Pheno151	IBS	5	0.148817107	0.073046779	0.041621489	1.1604607	1.005661387	1.33908801
Pheno558	IBS	5	-0.16333644	0.076180719	0.032027653	0.8493054	0.73150542	0.986075625
Pheno624	IBS	15	-0.124404	0.057831248	0.031463935	0.883023	0.788397206	0.989006124
Pheno882	IBS	15	-0.12071419	0.051504351	0.019090106	0.8862872	0.801185547	0.980428402

Supplementary Table S8. The results of MVMR anaysis for significant forward results.

**Exposure**	**Outcome**	**Beta**	**Se (standard error)**	***P* value**	**OR**	**Low_CI 95% of OR**	**Up_CI 95% of OR**
IBS	Pheno101	0.0082127	0.097064909	0.9325708	1.008246558	0.833572597	1.219523201
Major depression	Pheno101	0.2121933	0.077896688	0.061905252	0.729279026	0.546571829	1.973061305
Anxiety disorders	Pheno101	-0.07699	0.087393057	0.378335894	0.925898774	0.780140882	1.098889393
Insomnia	Pheno101	-0.060567	0.039421078	0.124437266	0.941230673	0.871244747	1.016838475
IBS	Pheno286	-0.175811	0.1066952	0.099395515	0.838776148	0.680495542	1.033872204
Major depression	Pheno286	-0.000686	0.085625741	0.993607469	0.999314207	0.844920718	1.181920225
Anxiety disorders	Pheno286	0.0913264	0.096063504	0.341762843	1.095626569	0.90759407	1.322615053
Insomnia	Pheno286	0.1077903	0.04333255	0.549822302	1.091092412	0.81992165	1.451946843
IBS	Pheno899	-0.204993	0.093072982	0.027630481	0.814653328	0.678808879	0.977683211
Major depression	Pheno899	0.1187853	0.074694386	0.111770749	1.126128118	0.972762136	1.30367383
Anxiety disorders	Pheno899	-0.025382	0.083800266	0.761974153	0.974937208	0.827264558	1.148970484
Insomnia	Pheno899	-0.001066	0.037799906	0.977507083	0.998934821	0.927601031	1.075754278
